# [^18^F]ML-10 PET imaging fails to assess early response to neoadjuvant chemotherapy in a preclinical model of triple negative breast cancer

**DOI:** 10.1186/s13550-019-0587-5

**Published:** 2020-01-06

**Authors:** Elodie Jouberton, Sébastien Schmitt, Emmanuel Chautard, Aurélie Maisonial-Besset, Marie Roy, Nina Radosevic-Robin, Jean-Michel Chezal, Elisabeth Miot-Noirault, Yann Bouvet, Florent Cachin

**Affiliations:** 1 Service de Médecine Nucléaire, Centre Jean Perrin, Clermont-Ferrand, France; 2 Département de Pathologie, Centre Jean Perrin, Clermont-Ferrand, France; 3 Université Clermont Auvergne, INSERM, Imagerie Moléculaire et Stratégies Théranostiques, UMR1240, Clermont-Ferrand, France; 4 Zionexa, Aubière, France; 5 Centre de Lutte Contre le Cancer, Centre Jean Perrin, 58 rue Montalembert, 63011 Clermont-Ferrand, France

**Keywords:** [^18^F]ML-10, apoptosis, PET imaging, triple negative breast cancer, in vivo, chemotherapy

## Abstract

**Purpose:**

Pathological complete response to the neoadjuvant therapy (NAT) for triple negative breast cancer (TNBC) is predictive of prolonged patient survival. Methods for early evaluation of NAT efficiency are still needed, in order to rapidly adjust the therapeutic strategy in case of initial non-response. One option for this is molecular imaging of apoptosis induced by chemotherapy. Therefore, we investigated the capacity of [^18^F]ML-10 PET imaging, an apoptosis radiotracer, to detect tumor cell apoptosis and early predict the therapeutic response of human TNBC.

**Results:**

Initially, the induction of apoptosis by different therapies was quantified. We confirmed, in vitro, that paclitaxel or epirubicin, the fundamental cytotoxic drugs for breast cancer, induce apoptosis in TNBC cell lines. Exposure of TNBC models MDA-MB-231 and MDA-MB-468 to these drugs induced a significant increase (*p* < 0.01) of the apoptotic hallmarks: DNA fragmentation, membrane phospholipid scrambling, and PARP activation. Secondarily, apoptotic fraction was compared to the intracellular accumulation of the radiotracer. [^18^F]ML-10 accumulated in the apoptotic cells after 72 h of treatment by paclitaxel in vitro; this accumulation positively correlated with the apoptotic fraction. In vivo, [^18^F]ML-10 was rapidly cleared from the nontarget organs and mainly eliminated by the kidneys. Comparison of the in vivo [^18^F]FDG, [^18^F]FMISO, and [^18^F]ML-10 uptakes revealed that the tumor accumulation of [^18^F]ML-10 was directly related to the tumor hypoxia level. Finally, after the in vivo treatment of TNBC murine xenografts by paclitaxel, apoptosis was well induced, as demonstrated by the cleaved caspase-3 levels; however, no significant increase of [^18^F]ML-10 accumulation in the tumors was observed, either on day 3 or day 6 after the end of the treatment.

**Conclusions:**

These results highlighted that PET imaging using [^18^F]ML-10 allows the visualization of apoptotic cells in TNBC models. Nevertheless, the increase of the chemotherapy-induced apoptotic response when using paclitaxel could not be assessed using this radiotracer in our mouse model.

## Background

Triple negative breast cancer (TNBC) is an aggressive disease that accounts for 15–20% of breast cancers and affects mainly young women [1]. This pathology is called “triple negative” due to the absence or very low expression of hormone receptors (for estrogen and for progesterone) by tumor cells, associated with a lack of amplification of the HER2 gene (*ERBB2*), all three biomarkers being the cornerstones of breast cancer therapy. Standard treatment of TNBC is currently based on neoadjuvant chemotherapy (NAT) using the FEC-taxane regimens. Pathological complete response (pCR) to NAT is associated with better overall survival [2]. One of the most important challenges in TNBC management is to develop more effective NAT, to allow for stronger early reduction of tumor mass, which prevents the appearance of sub-resistant clones responsible for metastatic disease [3, 4].

In such context, molecular imaging using 2-deoxy-2-[^18^F]fluoro-d-glucose ([^18^F]FDG) has been recently proposed for early assessment of the final TNBC response to NAT. Groheux et al. have demonstrated, in 78 TNBC patients, that the reduction of the [^18^F]FDG uptake (ΔSUVmax), assessed by positron emission tomography (PET) performed after only two cycles of NAT, highly correlates to pCR. However, in a recent meta-analysis which included 920 patients with breast cancers from 19 studies, the sensitivity and specificity of [^18^F]FDG PET/CT in predicting pCR were respectively of 84% and 66% [5, 6]. In around 20% of metastatic breast cancer patients, early recurrences have been reported in spite of treatment-induced total reduction of [^18^F]FDG uptake in the metastatic deposits. Numerous reasons for the [^18^F]FDG false negativity (FN) or positivity (FP) have been described, like incapacity to detect cancer cells in low number or with low glucose metabolism (FN), turn-off of glycolysis without subsequent cell death (FN) or presence of intense inflammatory infiltrate with high metabolism (FP) [7, 8]. Consequently, combination of the data collected by two different types of PET imaging tools, those that allow metabolic activity profiling and those that allow therapy-induced cell death assessment, would be of interest for predicting early response to NAT.

Apoptosis, known as the main type of chemotherapy-induced cell death, has been studied mainly over the past two decades using various microscopic and macroscopic imaging modalities [9, 10]. Monitoring apoptosis during treatment in the whole tumor is nowadays possible with the emergence of new nuclear imaging techniques. Several radiotracers have been developed to measure apoptosis in vivo such as phosphatidylserine (PS) exposure (annexin V radiolabeled with technetium-99m or fluorine-18), activation of caspases (isatins derivatives radiolabeled with fluorine-18) or mitochondrial outer membrane permeabilization ([^18^F]fluorobenzyl triphenylphosphonium cation) [11–13]. Among these radiolabeled compounds, [^99m^Tc]hydrazinonicotinamide-annexin V ([^99m^Tc]HYNIC-annexin V) has been used for clinical single photon emission computed tomography (SPECT) imaging of response to treatment of many solid tumors [14, 15]. The results of these clinical trials have revealed a strong affinity of the radiotracer for PS, and a correlation between the tumor radiotracer uptake and the number of dying cells. In another study, patients with malignant lymphoma, leukemia, non-small cell lung carcinoma, or head and neck squamous cell carcinoma were enrolled in a clinical trial to follow their therapeutic response with [^99m^Tc]HYNIC-annexin V [16]. An increase of [^99m^Tc]HYNIC-annexin V tumor uptake after therapy correlated with clinical outcome. However, even if this radiotracer holds promise for future nuclear imaging of apoptosis, it is currently not used in clinical routine due the difficulty to discriminate apoptotic from necrotic cells [17].

Recently, pre-clinical experiments and clinical trials have been conducted using the 2-(5-[^18^F]fluoropentyl)-2-methylmalonic acid ([^18^F]ML-10)**,** an apoptosis pH-sensitive radiotracer developed by ApoSense Ltd. (Petach-Tikva, Israel). These studies have demonstrated the ability of this small molecule (206 Da) to specifically target apoptotic cells, while being excluded from viable or necrotic cells [18].

The [^18^F]ML-10 radiotracer is highly stable in vivo and presents a rapid clearance from non-targeted organs and a favorable dosimetry profile [19]. Several clinical trials have recently evaluated [^18^F]ML-10 for early detection of response to radiation therapy in patients with brain metastases [20, 21]. In those studies, early treatment-induced changes in the [^18^F]ML-10 tumor accumulation were measured by voxel-based analysis and correlated with changes in anatomical dimensions, as visualized with magnetic resonance imaging (MRI). The authors confirmed the value of PET imaging with [^18^F]ML-10 for early assessment of tumor response to therapy as well as the potential of this radiotracer to visualize apoptosis. In addition, studies in two types of solid tumors (nasopharyngeal carcinoma and head/neck carcinoma) have also highlighted the capacity of the [^18^F]ML-10 radiotracer to target apoptotic cells following cancer chemotherapy [22, 23]. However, no pre-clinical or clinical studies have been conducted in TNBC models.

In this study, we first investigated the induction of apoptosis by drugs currently used in NAT (paclitaxel, epirubicin) on in vitro and in vivo TNBC models. Apoptosis was evaluated by multiple strategies including western blotting and flow cytometry. We then explored the capacity of [^18^F]ML-10 to target apoptosis in TNBC models. We applied longitudinal PET imaging to monitor the dynamics of apoptotis induced by paclitaxel. The input of [^18^F]ML-10 imaging to classical [^18^F]FDG and [^18^F]FMISO PET imaging was also assessed. As [^18^F]ML-10 is a pH-sensitive molecule, the impact of extracellular pH variation on radiotracer uptake was finally evaluated.

## Methods

### TNBC cell lines and culture

Human epithelial breast cancer cell lines, MDA-MB-231 (ATCC® HTB-126) and MDA-MB-468 (ATCC® HTB-132™), were purchased from American Type Culture Collection (ATCC, Manassas, USA) and cultured in Eagle’s MEM medium (Gibco®) supplemented with 1 mM l-glutamine, 1 mM sodium pyruvate, 1 mM non-essential amino acids, 4 μg/mL gentamycin and 10% fetal calf serum (Dutscher, Brumath, France). Cells were maintained at 37 °C in a humidified atmosphere containing 5% CO_2_.

### [^18^F]ML-10 uptake assay of TNBC cell lines treated by paclitaxel

After induction of apoptosis by paclitaxel treatment, cells were incubated with 0.37 MBq of [^18^F]ML-10 for 15 min in Hank’s Balanced Salt Solution (HBSS, Gibco®) supplemented with 10 mM HEPES buffer at 37 °C. The [^18^F]ML-10 uptake was terminated by three cycles of washing with the HBSS-HEPES mixture, followed by centrifugation. Cell pellets were recovered, and radioactivity was measured using a gamma counter (Wizard 1480, Perkin Elmer). The cellular uptake was presented as the radioactivity amount per 10^6^ cells (MBq/10^6^ cells). [^18^F]ML-10 uptake was correlated with the apoptotic hallmarks of annexin-V binding.

To induce pH variations, cells treated and untreated were centrifuged and re-suspended in HBSS-HEPES, buffered at different pH values, ranging from 5.9 to 8.0.

### Animal model

All animal studies were conducted in accordance with the Guide for the Care and Use of Laboratory Animals published by the US National Institutes of Health (NIH Publication n°85–23, revised 1996) and with the relevant guidelines and regulations approved by both the local Ethic Committee of Clermont-Ferrand (France) and the French Ministry of Education and Research (approval n°18284-2018122716575316). One hundred and ten female NMRI Nude (Crl: NMRI-Foxn1nu) mice of 6–7 weeks old were purchased from Janvier Laboratory (Le Genest-Saint-Isle, France) and housed under environmentally controlled conditions with free access to standard food and water. Tumor xenografts were induced by subcutaneous injection, into the right front leg of the mice, of 10^6^ cells suspended in 75 μL of PBS. Orthotopic implantation was performed by inoculating 10^5^ cells suspended in 25 μL of PBS into the second thoracic mammary fat pad. Tumor volumes were determined by using the following formula: TV (mm^3^) = [length × (width)^2^]/2. Variation change in tumor volume was calculated by using the following formula: Δ tumor volume (%) = [(TV_dx_ − TV_d0_)/TV_d0_] × 100 with dx representing day of acquisition PET and d0 representing acquisition before treatment.

### PET imaging experiments with [^18^F]ML-10, [^18^F]FMISO, and [^18^F]FDG

Images focused on the tumor were performed using a small-animal PET device (eXplore VISTA; GE Healthcare). Mice were anesthetized with 1.5–2% isoflurane/oxygen (1 L/min) and maintained during image acquisition. Dynamic PET imaging was performed immediately (0–1 min) after i.v. injection of 22–30 MBq of [^18^F]ML-10. List mode data were collected for 45 min and performed 4 weeks after tumor implantation for MDA-MB-468 xenografts and 3 weeks for MDA-MB-231 xenografts. Static PET imaging, focused on the tumor, was performed at 30 min ([^18^F]ML-10), 1 h ([^18^F]FDG), and 4 h ([^18^F]FMISO) after i.v. injection of 11–18 MBq of the corresponding radiotracers. Each acquisition lasted 15 min. In addition, mice receiving [^18^F]FDG were fasted 12 h before radiotracer injection. The energy window used was 250–700 keV and the spatial resolution of this system was 1.4 mm. Images were reconstructed according to the OSEM 2D algorithm including corrections for scanner dead time, scattered radiations, and randoms but no correction of attenuation. Regions of interest were drawn on images of the tumor and the kidneys, liver, heart, and muscle of the contralateral paw, at each imaging time. Time-activity curves were presented as a mean of standard uptake value (%DI/g). For tumor analysis, the max and mean of standard uptake value (SUVmax and SUVmean) were measured and tumor/muscle ratios were calculated. For studies concerning the correlation between radiotracer accumulations, the same mice were imaged with the three radiotracers at 1-day intervals.

After PET imaging protocols, at 60 min post-injection of [^18^F]ML10, urine was collected and the percentage of unchanged [^18^F]ML-10 radiotracer was determined by analytical radio-HPLC and radio-TLC as described in Additional file 1.

### Study design for assessment of early therapeutic response to NAT

**Fig. 1:**
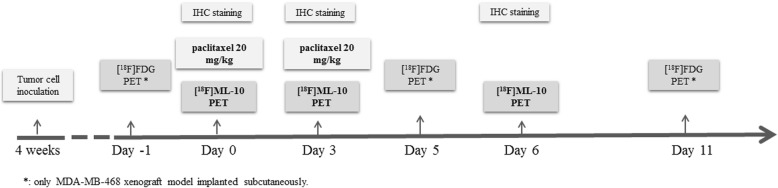
Study design with small-animal PET imaging of a murine breast tumor model by subcutaneous or orthotopic implantation. IHC indicates immunohistochemical and PET indicates positron emission tomography acquisition

When tumors have reached an average volume of 50 ± 16 mm^3^, the mice were randomized in two groups. One group was injected of 0.9% NaCl (control group), and another group was given a therapeutic protocol that consisted of two paclitaxel doses (20 mg/kg), at a 72-h interval, injected via the tail vein. Treatment was performed at day 0 (d0), after baseline [^18^F]ML-10 PET (Fig. 1). Subsequent [^18^F]ML-10 PETs were performed at d0, d3, and d6 post-treatment. Sequential [^18^F]FDG PETs were performed 1 day before treatment (d-1) and after treatment (d5 and d11) only for the MDA-MB-468 subcutaneous model. In a parallel experiment, two groups of 18 mice were considered for apoptosis evaluation by immunofluorescence for cleaved caspase-3.

### Statistical analysis

All values were presented as the mean ± standard deviation (SD) and were statistically analyzed with GraphPad Prism (version 5.0 GraphPad Software, Inc., San Diego, CA). Each parameter in vitro was compared between untreated and treated groups by the Mann-Whitney test. Pearson correlation analyses were performed to assess the correlation between the [^18^F]ML-10 uptake and the percentage of apoptotic cells or extracellular pH. Each parameter in vivo was compared between control and paclitaxel groups by the Mann-Whitney test. Pearson correlation analyses were performed to assess the correlation between the [^18^F]ML-10 SUVmean and tumor volume or to compare radiotracers SUVmean. For each radiotracer, Pearson correlation analyses were performed to assess the correlation between the SUVmean and SUVmax. *p* was considered statistically significant, when < 0.05 (*p < 0.05, **p < 0.01, ***p < 0.001).

For more details about assessment of apoptosis and radiosyntheses of [^18^F]ML-10, [^18^F]FDG, and [^18^F]FMISO, see Additional file 1.

## Results

### Epirubicin and paclitaxel induce apoptosis in TNBC cell lines

Cytotoxic activity of epirubicin and paclitaxel was first evaluated in two TNBC cell lines. IC_50_ values of paclitaxel and epirubicin were, respectively 3.5, ± 0.5, and 25 ± 0.02 nM for MDA-MB-468 cells, and 0.02 ± 0.01 μM and 1.54 ± 0.02 μM for MDA-MB-231 cells (Table 1).

**Table 1:** IC_50_ (μM) values for MDA-MB-468 and MDA-MB-231 cell culture. Results are shown as mean ± SD. (*n* = 3 independent experiments)

Cell lines	MDA-MB-231	MDA-MB-468
Epirubicin	1.54 ± 0.02	0.025 ± 0.0002
Paclitaxel	0.02 ± 0.007	0.0035 ± 0.0005

**Fig. 2:**
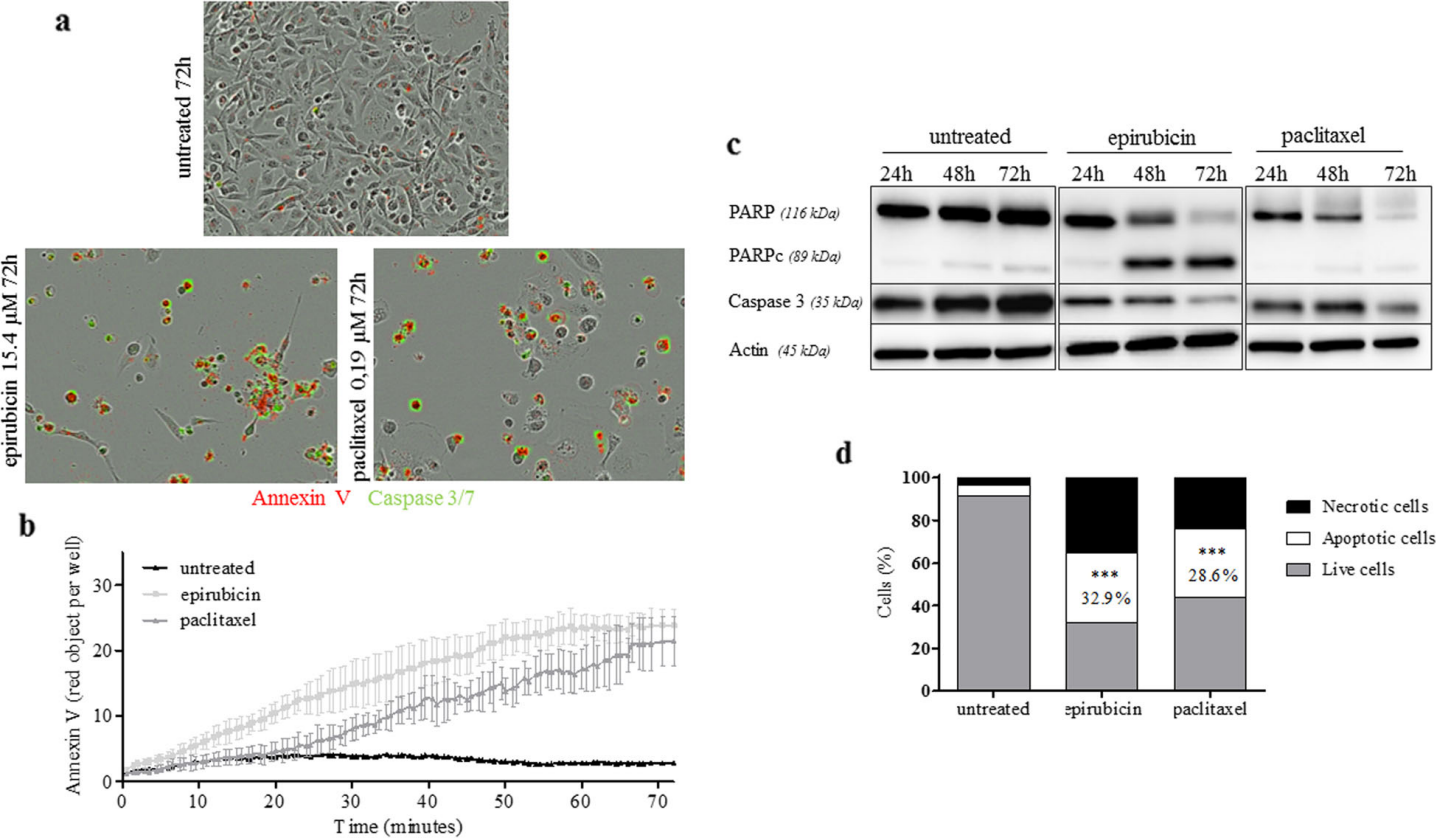
Apoptotic induction after treatment by epirubicin or paclitaxel in MDA-MB-231 cells. **a** Time-lapse images detection of apoptosis in MDA-MB-231 cells in the presence of Caspase-3/7 Green Apoptosis reagent and Annexin V Red Reagent. **b** Percent of apoptotic cells with high annexin V reagent was assessed in time-lapse imaging (IncuCyte) over 72 h. **c** Western blot analysis showing the cleavage of PARP and Caspase 3 decrease. Actin was used as a loading control. **d** Quantitative analysis of apoptotic cells measured by flow cytometry in untreated, epirubicin-treated, or paclitaxel-treated MDA-MB-231 cells for 72 h. (*n* = 3 independent experiments; mean ± SD). Compared to untreated, ***p < 0.001 (Mann-Whitney test)

Apoptosis was induced in the two human TNBC cell lines by treatment with paclitaxel or epirubicin at a 10-fold IC_50_ dose. Under treatments, blended phase-contrast and red/green images showed an increase of annexin V and cleaved caspase 3/7 expression in the treated cells compared to the untreated cells (Fig. 2). Real-time quantification of the annexin V revealed an increase of apoptosis in the treated cells compared to the untreated cells at 72 h post-treatment: 2.8 ± 3.1 red objects per well for control, and 23.8 ± 2.4 and 21.4 ± 3.7 red objects per well for epirubicin and paclitaxel-treated cells respectively. Western blotting analysis performed at 24, 48, and 72 h post-treatment showed a decrease of caspase 3 and an increase of cleaved PARP expression at the end of each post-treatment interval, with a peak at 72 h. An increase of apoptotic cell number was confirmed by flow cytometry, 72 h after treatment by epirubicin (32.9 ± 1.2%) or paclitaxel (28.6 ± 7.9%) compared to untreated cells (3.3 ± 1.9%) (Table 2 and 3).

**Table 2:** Apoptosis analysis of MDA-MB-231(a) and MDA-MB-468(b) cells after incubation with treatment at 10xIC_50_ for 24 h, 48 h, and 72 h by flow cytometry using Annexin V/PI apoptosis assay. Results are shown as mean ± SD (*n* = 3 independent experiments)

MDA-MB-231							
	Untreated			Epirubicin	Paclitaxel		
	24 h	48 h	72 h	72 h	24 h	48 h	72 h
Live cells (%)	93.8 ± 2.5	94.5 ± 1.7	94.7 ± 2.2	2.6 ± 1.3	71.8 ± 6.8	59.9 ± 8.0	50.7 ± 13.3
Apoptotic cells (%)	3.3 ± 1.9	3.4 ± 0.4	2.0 ± 0.9	32.9 ± 1.2	10.5 ± 2.0	18.6 ± 2.5	28.6 ± 7.9
Necrotic cells (%)	2.9 ± 0.9	5.1 ± 1.4	3.2 ± 1.3	64.4 ± 1.7	23.7 ± 1.6	21.5 ± 6.6	20.7 ± 6.2

**Table 3:** Apoptosis analysis of MDA-MB-231(a) and MDA-MB-468(b) cells after incubation with treatment at 10xIC_50_ for 24 h, 48 h, and 72 h by flow cytometry using Annexin V/PI apoptosis assay. Results are shown as mean ± SD (*n* = 3 independent experiments)

MDA-MB-468							
	Untreated			Epirubicin	Paclitaxel		
	24 h	48 h	72 h	72 h	24 h	48 h	72 h
Live cells (%)	88.7 ± 1.4	83.3 ± 2.4	83.8 ± 2.3	30.1 ± 0.9	67.6 ± 2.5	45.3 ± 3.2	23.0 ± 1.6
Apoptotic cells (%)	6.8 ± 1.1	8.6 ± 2.3	*8.8 ± 0.7*	49.5 ± 1.2	23.6 ± 1.5	38.8 ± 2.8	53.3 ± 4.2
Necrotic cells (%)	4.5 ± 1.6	8.1 ± 3.0	7.3 ± 2.7	20.4 ± 0.3	8.8 ± 2.0	15.9 ± 3.7	23.8 ± 4.3

**Fig. 3:**
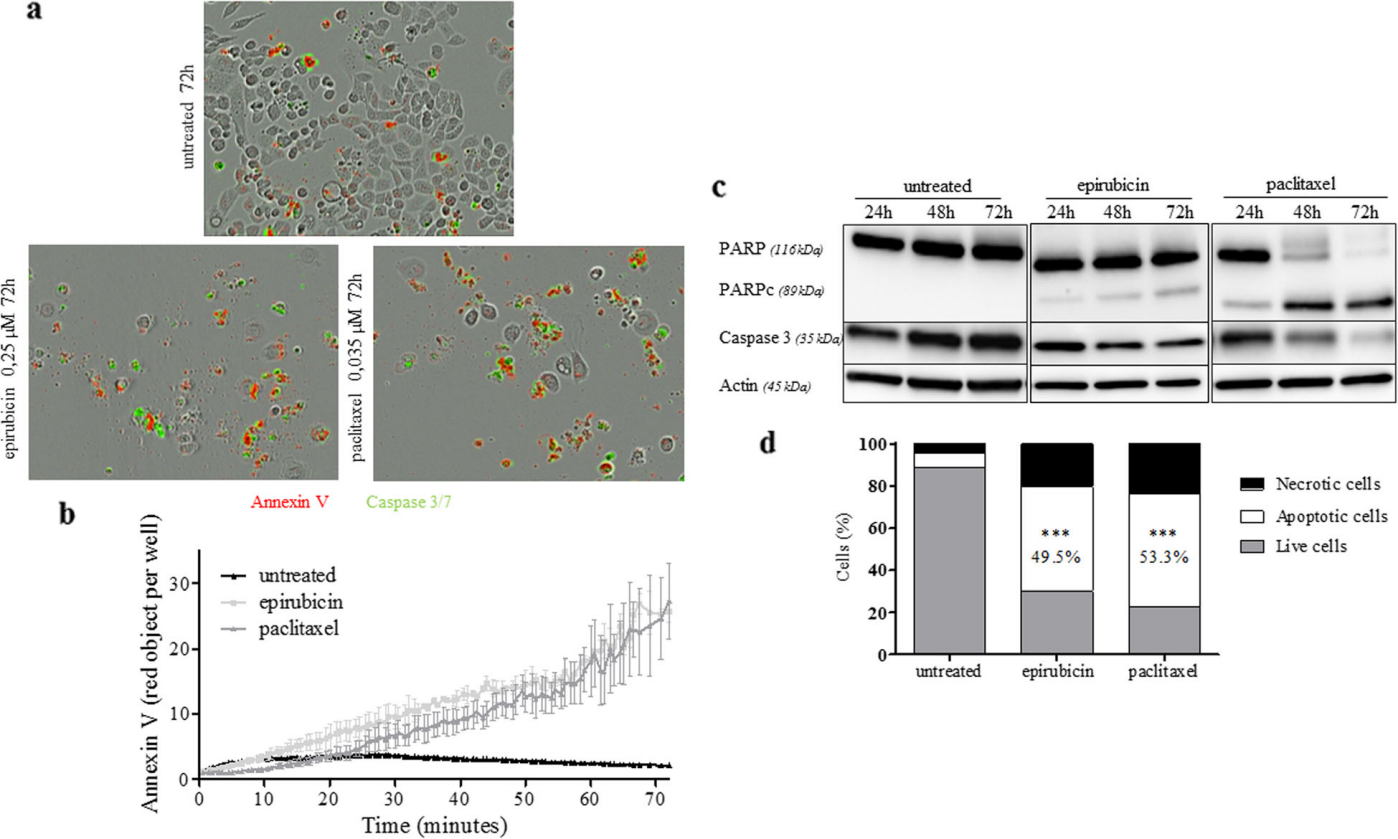
Apoptotic induction after treatment by paclitaxel or epirubicin in MDA-MB-468 cells. **a** Time-lapse images detection of apoptosis in MDA-MB-468 cells in the presence of Caspase-3/7 Green Apoptosis reagent and Annexin V Red Reagent. **b** Percent of apoptotic cells with high annexin V reagent was assessed in time-lapse imaging (IncuCyte) over 72 h. **c** Western blot analysis showing the cleavage of PARP and Caspase 3. Actin was used as a loading control. **d** Quantitative analysis of apoptotic cells measured by flow cytometry in untreated, epirubicin-treated, or paclitaxel-treated MDA-MB-468 cells for 72 h. (*n* = 3 independent experiments; mean ± SD). Compared to untreated, ***p < 0.001 (Mann-Whitney test)

MDA-MB-468 cells presented more staining in untreated cells compared to MDA-MB-231, as seen on the real-time image (Fig. 3). These findings were confirmed by western blotting analysis performed at 24, 48, and 72 h. We also observed a decrease of caspase 3 and an increase of cleaved PARP abundance, with a peak at 72 h post-treatment. Moreover, flow cytometry studies showed a higher percentage of apoptotic cells after 72 h of treatment (49.5 ± 1.2% for epirubicin, 53.3% ± 4.2% for paclitaxel) than in the untreated cells (6.8 ± 1.1%) (Table 2 and 3).

Thus, these two cell lines showed different response to treatments, with the biggest apoptotic fraction induced in the MDA-MB-468 cells treated by paclitaxel. Therefore, those cells were used in the radiotracer uptake studies.

### Paclitaxel induces apoptosis in murine xenografts of TNBC cell lines

**Fig. 4:**
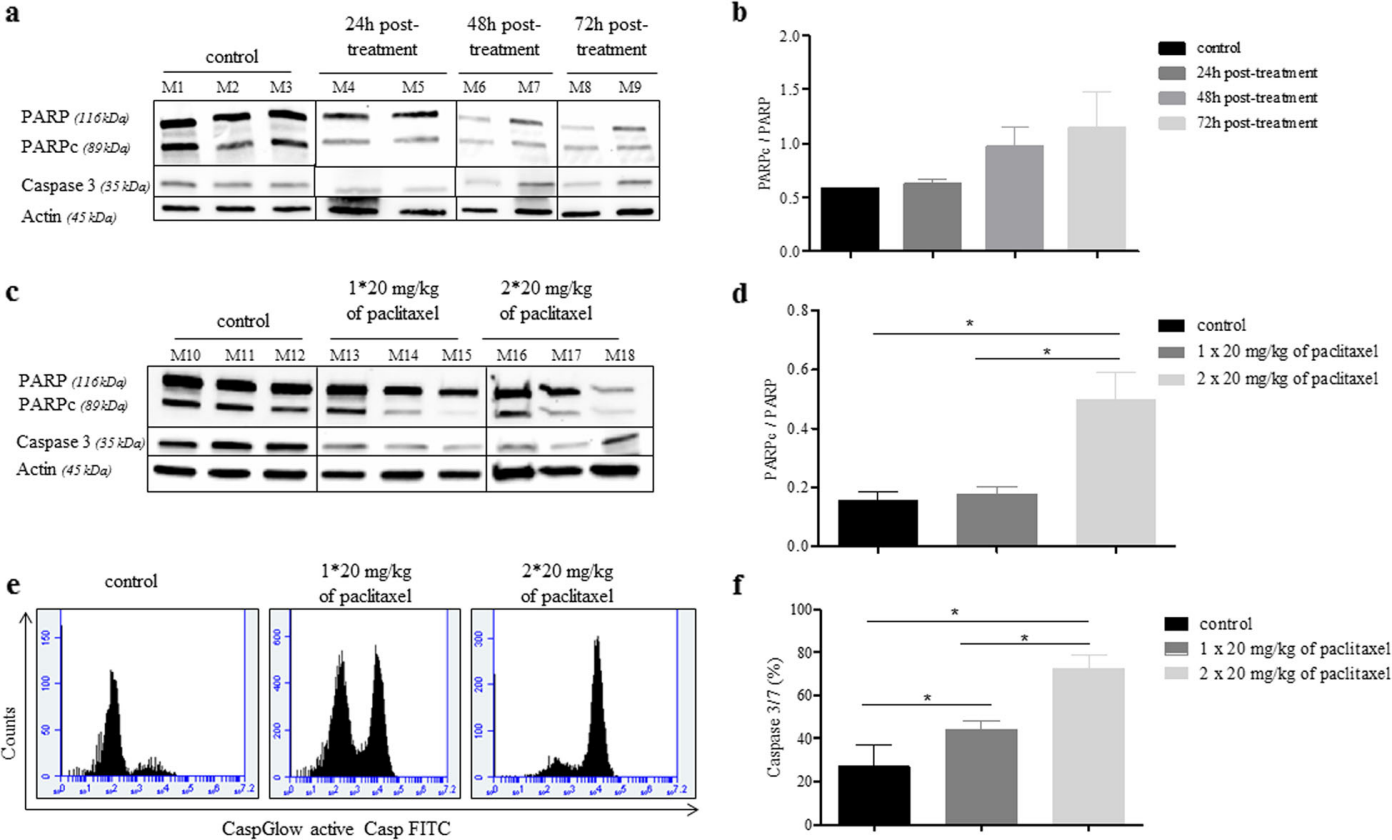
Characterization of apoptotic tumor after treatment by paclitaxel in MDA-MB-468 subcutaneously xenograft model. **a–c** Western blot analysis showing the cleavage of PARP and Caspase 3 on tumors samples. Actin was used as a loading control. Individual bands (M1, M2, M3) correspond to 2–3 independent tumor samples per group of treatment. **b–d** Quantification of cleaved PARP as a ratio to PARP. Data are presented as the mean ± SD. Mann-Whitney test was performed (*p < 0.05). Significant difference versus control group. **e** Increase of cleaved caspase 3-positive tumor cells was observed after paclitaxel treatment. **f** Quantitative analysis of apoptotic cells measured by flow cytometry in control or paclitaxel treated. Data are presented as the mean ± SD. Compared to control, ***p < 0.001 (Mann-Whitney test)

First, we characterized the induction of apoptosis in a MDA-MB-468 subcutaneously xenograft model at 24, 48, and 72 h after the injection of one dose of paclitaxel at 20 mg/kg. Western blotting analysis evidenced a decrease of caspase 3 and an increase of cleaved PARP from 24 h with a maximum at 72 h (*p* = 0.1 at 72 h; Fig. 4a–b). Then, we characterized apoptosis after two doses of 20 mg/kg paclitaxel at a 72-h interval and observed a decrease of caspase 3 as well as a significant increase of cleaved PARP to control (*p* = 0.028) and to the one dose group (*p* = 0.028; Fig. 4c, d).

To confirm the specificity of labeling of apoptotic cells, we analyzed the percentage of cleaved caspase 3 cells by flow cytometry. As shown in Fig. 4e, f, cleaved caspase 3 in the paclitaxel-treated tumors (72.4% ± 6.7%) was significantly higher than in the control group (27.0% ± 10.0%; *p* = 0.019).

### [^18^F]ML-10 accumulates in the apoptotic TNBC cells in vitro

**Fig. 5:**
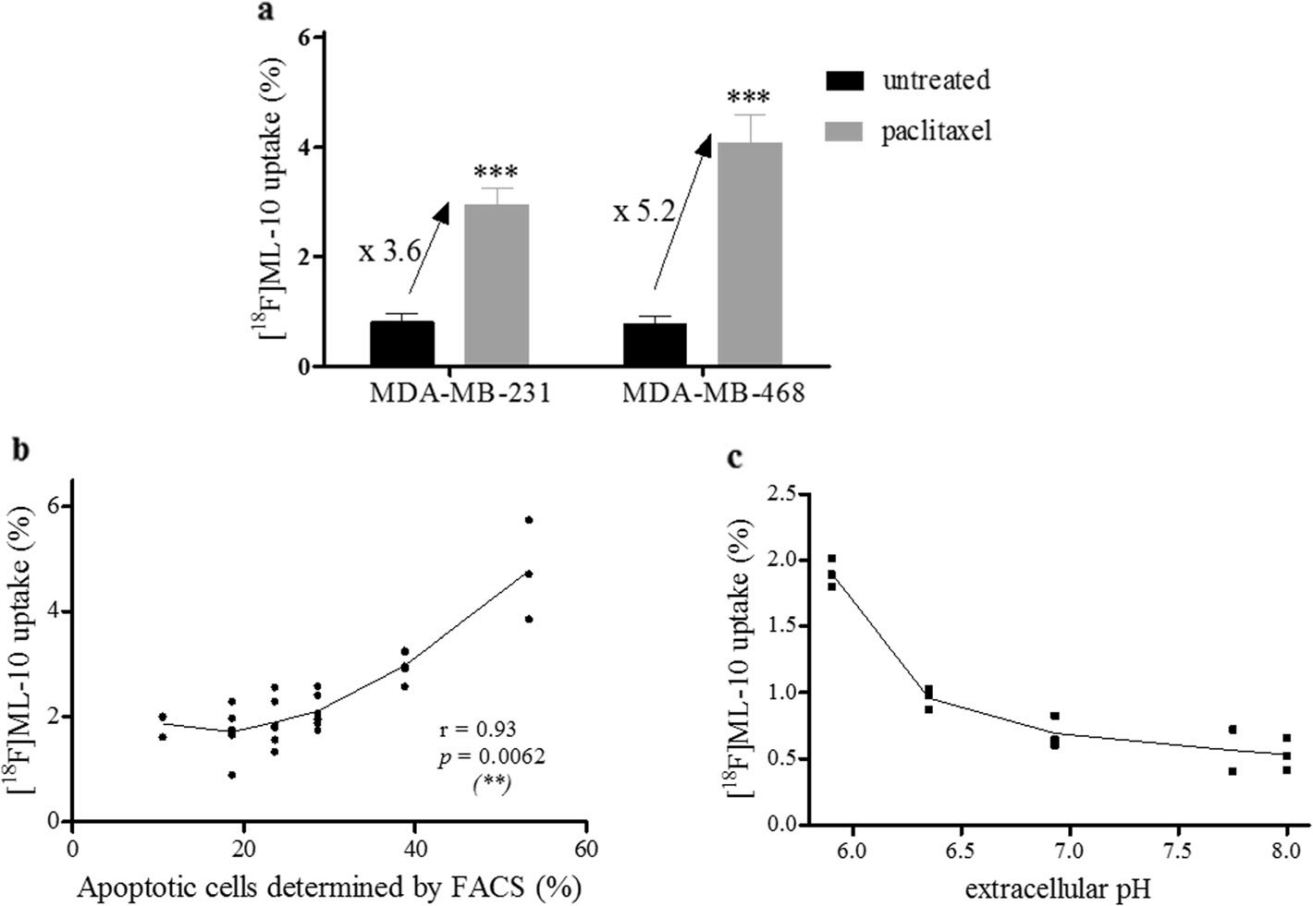
[^18^F]ML-10 uptake. **a** [^18^F]ML-10 was added to the cell suspension containing adherent cells 72 h after paclitaxel and incubation for 15 min and its uptake was determined. Results are shown as mean ± SD (*n* = 3 independent experiments). Mann-Whitney test was performed (***p < 0.001). **b** Correlation between the percentage of apoptotic cells and [^18^F]ML-10 binding amount in paclitaxel-treated MDA-MB-468 cells. The percentage of cells in apoptosis was determined using Annexin V/PI staining and expressed as the proportion of Annexin V-positive cell counts in total counts. Pearson correlation was performed (**p < 0.01). **c** Effect of acidification on [^18^F]ML-10 uptake. pH dependence of [^18^F]ML-10 uptake, with an extracellular pH ranging from of 5.9 to 8.0.

Experiments were carried out in adherent cells (which contain the apoptotic cells and viable cells), after 72 h of treatment by 0.035 μM of paclitaxel only. [^18^F]ML-10 uptake increased from 0.8 ± 0.4% and 0.8 ± 0.3% at the baseline to 2.9 ± 0.8% and 4.1 ± 1.5% at 72-h post-treatment, respectively for MDA-MB-231 and MDA-MB-468 cells. [^18^F]ML-10 uptake was 3.6 and 5.2 times higher in the treated cells compared to the untreated cells, in MDA-MB-231 and MDA-MB-468 respectively (*p* < 0.001; Fig. 5a).

Furthermore, the sensitivity of the radiotracer [^18^F]ML-10 to detect apoptotic cells was evaluated by comparing the radiotracer uptake with the percentage of cells in apoptosis post-treatment (Fig. 5b). [^18^F]ML-10 uptake was 1.9 ± 0.4% at 24-h post-treatment (23.6% of apoptotic cells) and 2.9 ± 0.2% at 48-h post-treatment (38.8% of apoptotic cells) for MDA-MB-468 cells. [^18^F]ML-10 uptake increased with the apoptotic fraction (*p* = 0.006).

All together, these results demonstrated that [^18^F]ML-10 detects cells in early apoptosis after paclitaxel-treatment in the TNBC cell lines investigated.

### [^18^F]ML-10 is sensitive to extracellular pH variations

Scrambling processes in early apoptosis reduce the pH of the external membrane leaflet and cytoplasm (acidification). To reproduce this effect in vitro, the pH of the culture medium was modified to evaluate the impact of extracellular pH on the ability of [^18^F]ML-10 to bind the apoptotic cells. Studies were conducted only on MDA-MB-468 cells after 72 h of treatment at a 10-fold IC_50_ dose of paclitaxel and incubation with [^18^F]ML-10 at pH 5.9 to 8.0. As shown in Fig. 5c, acidification of the medium increased the [^18^F]ML-10 uptake. At pH 8.0, the radiotracer uptake was only 60% of the uptake at neutral pH (7.0), whereas it was 100% higher at pH 6.0 than at the neutral pH. Consequently, the high dependence of the radiotracer uptake on the extracellular pH, in the physiological pH range, was confirmed.

### Imaging biokinetics and high in vivo stability of [^18^F]ML-10

**Fig. 6:**
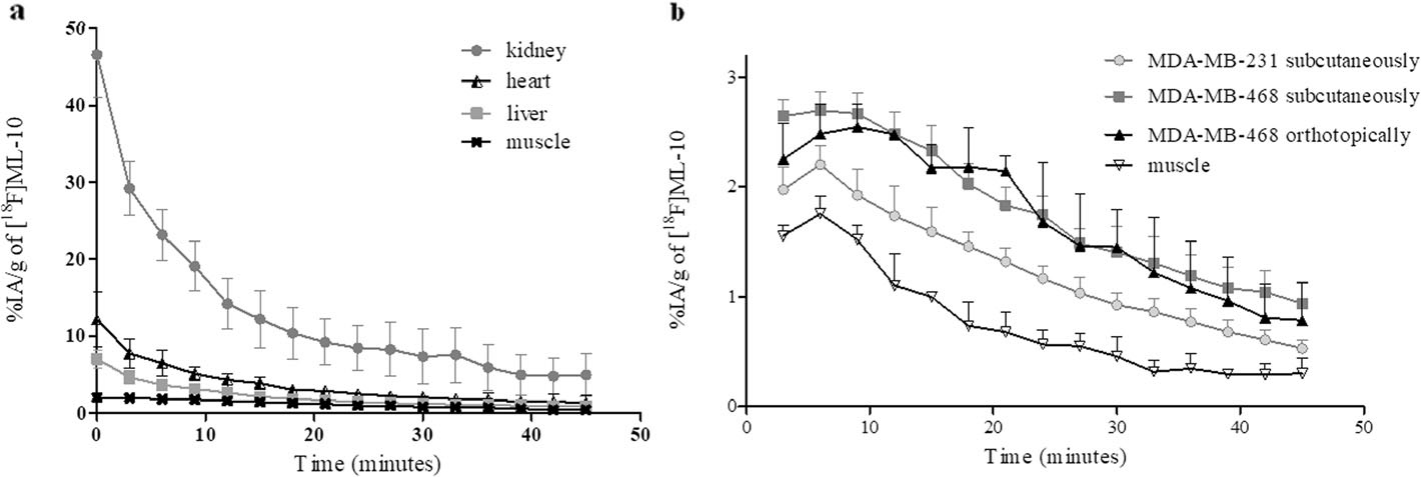
Time activity curves after bolus injection of 29.5 ± 4.1 MBq [^18^F]ML-10 of triple negative breast cancer models. **a** Time activity curves showing uptake of [^18^F]ML-10 in the kidneys, liver, heart, and muscle over 45 min (*n* = 6). **b** Time-activity curves showing uptake of [^18^F]ML-10 in muscle, MDA-MB-231 xenograft tumor (*n* = 3), MDA-MB-468 xenograft tumor (*n* = 3), and MDA-MB-468 orthotopic tumor (*n* = 3) over 45 min. Radioactivity was expressed as a percentage of the injected activity per gram of tissue (%IA/g). Data are presented as mean ± SD

For in vivo studies, we first evaluated the biokinetics of [^18^F]ML-10 in tumor-bearing mice with dynamic PET imaging. Average time-activity curves, for each xenograft models, revealed a rapid clearance of the radiotracer from the nontarget organs, through renal excretion (29.2 ± 3.5 %IA/g at 3 min, 12.2 ± 3.7 %IA/g at 15 min, and 7.3 ± 0.3 %IA/g at 30 min) (Fig. 6a). Analysis of urine samples, at 60 min after i.v. injection of [^18^F]ML-10, did not reveal any radioactive metabolites as shown by radio-HPLC and radio-TLC analyses ( Additional file 1: Figure S1). These results suggested a high in vivo stability of this radiotracer.

After evaluation of the whole-body biokinetics, the potential of [^18^F]ML-10 for the assessment of tumor apoptosis in TNBC xenografts (Fig. 6b) was explored. As expected, the distribution of the radiotracer in muscle decreased over time and was 0.76 ± 0.3 %IA/g at 30 min. In both MDA-MB-468 xenograft models, the radiotracer uptake in tumor reached a plateau from 1 to 12 min post-injection (p.i.) and was then eliminated from the tumor. This plateau was not observed in the muscle or in the MDA-MB-231 xenograft model, which showed tumor accumulation of the radiotracer was similar to the muscle and vascular background activity, demonstrating that the accumulation in the tumor was non-specific.

### [^18^F]ML-10 tumor accumulation correlates with the apoptotic fraction in TNBC xenografts

**Fig. 7:**
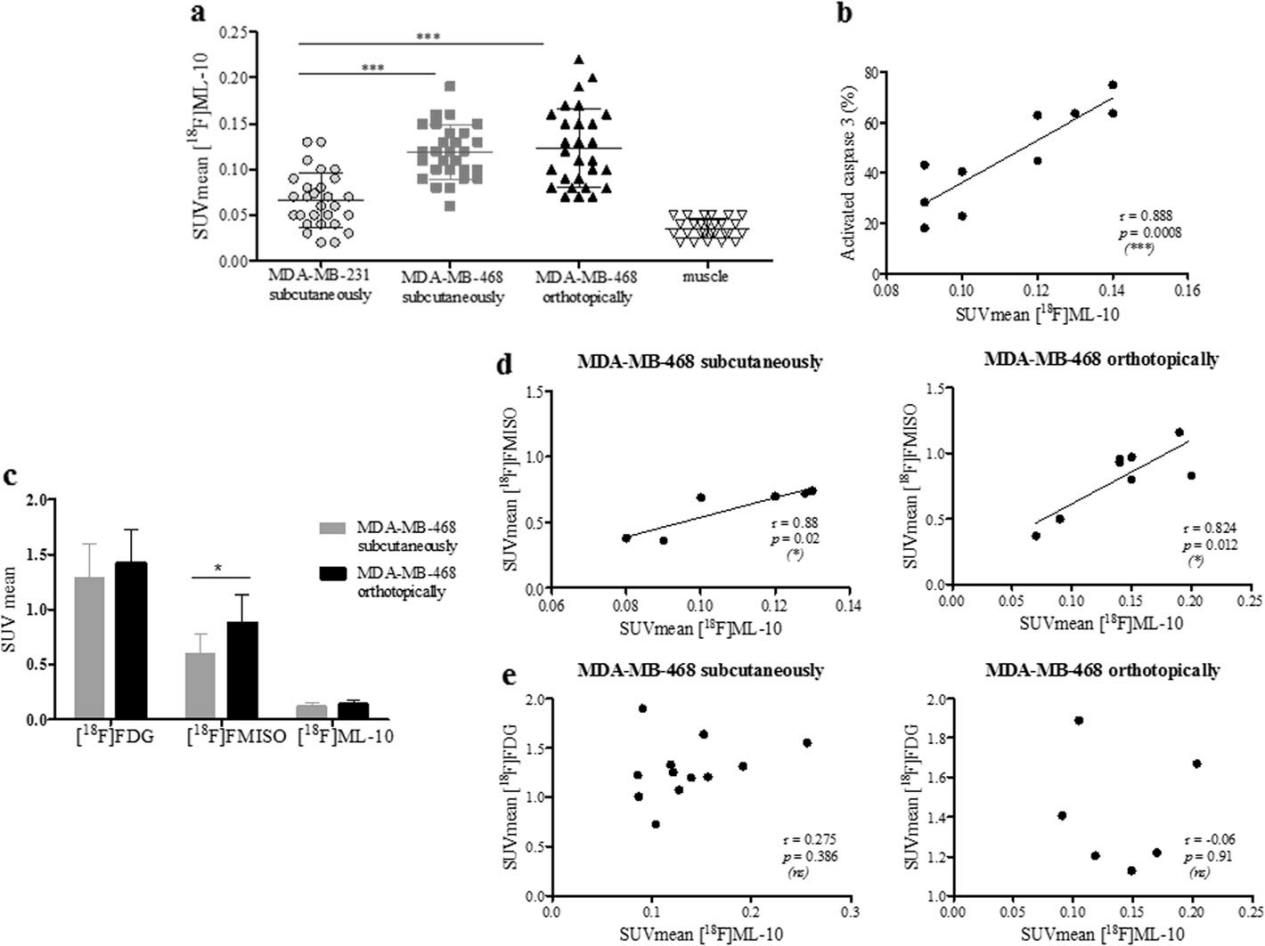
In vivo imaging analysis of baseline PET imaging. **a** SUVmean showing uptake of [^18^F]ML-10 in MDA-MB-231 subcutaneous xenograft tumor, MDA-MB-468 subcutaneous xenograft tumor, and MDA-MB-468 orthotopic xenograft tumor and muscle. The Mann-Whitney test was performed (***p < 0.001). **b** Flow cytometric quantitation of cell apoptosis was compared with cell-associated radioactivity for [^18^F]ML-10. Pearson correlation was performed (***p < 0.001). **c** SUVmean showing uptake of [^18^F]FDG, [^18^F]FMISO, and [^18^F]ML-10 in MDA-MB-468 subcutaneous or orthotopic xenograft tumor. The Mann-Whitney test was performed (*p < 0.05, **p < 0.01, ***p < 0.001). Correlation SUVmean of [^18^F]FDG **d** or [^18^F]FMISO **e** uptake in tumor with SUVmean of [^18^F]ML-10. Pearson correlation was performed (*p < 0.05)

Among the three investigated models (Fig. 7a and Additional file 1: Table S1), significantly lower [^18^F]ML-10 uptake was detected in the MDA-MB-231 xenografts (SUVmean = 0.06 ± 0.03), compared to the MDA-MB-468 subcutaneous model (SUVmean = 0.12 ± 0.03; *p* < 0.001) and orthotopic model (SUVmean = 0.15 ± 0.04; *p* < 0.001). The SUVmean of [^18^F]ML-10 positively correlated to the apoptotic tumor fraction determined by flow cytometry (Fig. 7b; *p* < 0.001). Interestingly, an increased [^18^F]ML-10 uptake also positively correlated with tumor volume in the subcutaneous MDA-MB-231 (*p* = 0.002) and MDA-MB-468 (*p* = 0.02) models but not in the MDA-MB-468 orthotopic model (*p* = 0.987; Additional file 1: Figure S2). Compared to the MDA-MB-231, a basal apoptotic fraction of 27 ± 10 % was detected by [^18^F]ML-10 PET imaging in the MDA-MB-468 model. In view of these results, [^18^F]ML-10 was only compared with [^18^F]FMISO and [^18^F]FDG on MDA-MB-468 xenograft models.

### Comparison of in vivo [^18^F]FDG, [^18^F]FMISO, and [^18^F]ML-10 uptake in a MDA-MB-468 model

Regarding the accumulation of the [^18^F]FDG, no difference between the orthotopic and the subcutaneous MDA-MB-468 model was observed (*p* = 0.54; Fig. 7c and Additional file 1: Table S2). On the other side, the orthotopic model showed a greater [^18^F]FMISO uptake (SUVmean = 0.81 ± 0.26) than the subcutaneous model (SUVmean = 0.60 ± 0.18; *p* = 0.04). A correlation between the [^18^F]ML-10 uptake and the level of hypoxia in the tumor, assessed by [^18^F]FMISO, was clearly evidenced in both the subcutaneous (*r* = 0.88, *p* = 0.02) and orthotopic (*r* = 0.82, *p* = 0.01) MDA-MB-468 xenograft models (Fig. 7d). No correlation was found between the accumulation of [^18^F]ML-10 and [^18^F]FDG in both xenograft models (Fig. 7e).

### [^18^F]ML-10 uptake, detected by PET imaging, doses not correlate with TNBC xenograft response to paclitaxel in subcutaneous model

**Fig. 8:**
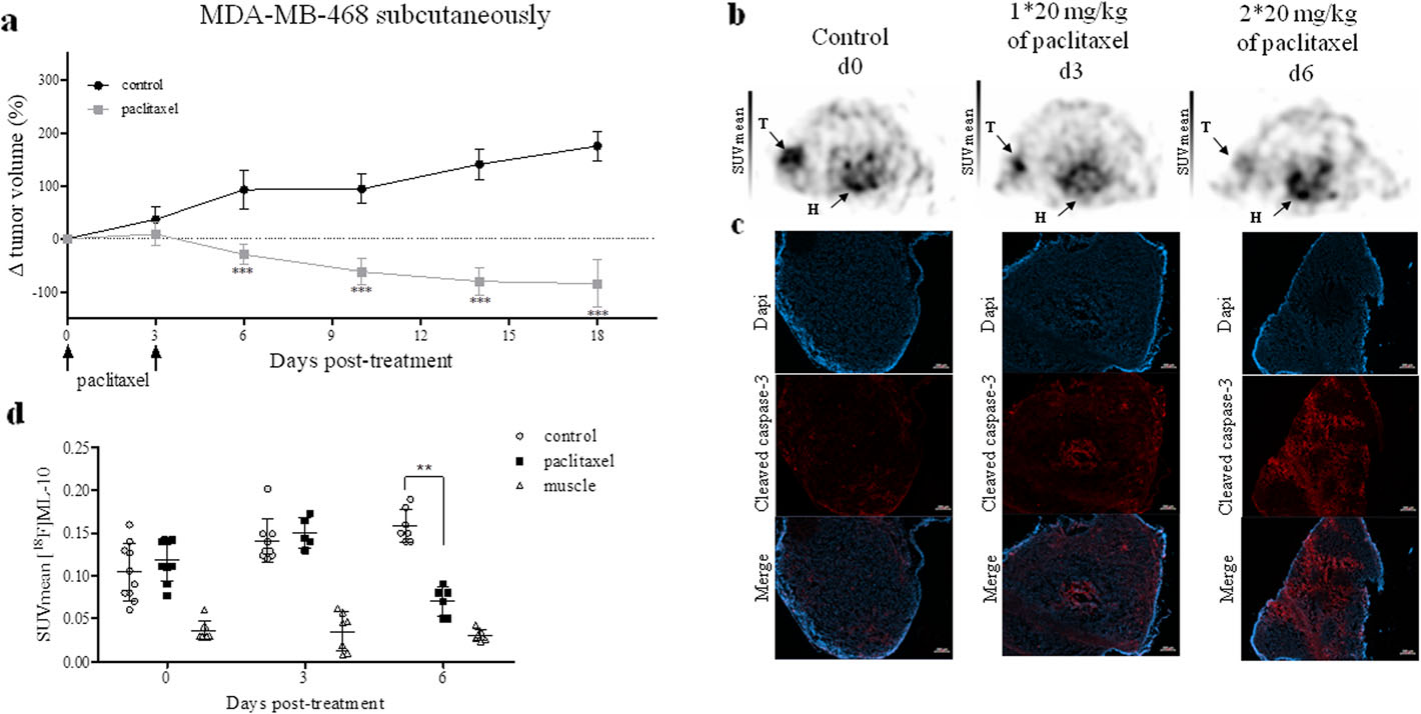
Evaluation of early response by [^18^F]ML-10 PET imaging on subcutaneously model. **a** Evaluation of tumor volume after two i.v. injections of paclitaxel (20 mg/kg) at a 72-h interval. Data are represented as mean ± SD. Compared to control, ***p < 0.001 (Mann-Whitney test). **b** Images of [^18^F]ML-10 PET of representative MDA-MB-468 subcutaneous xenograft model performed and 72-h post-treatment. T represented tumor and H represented heart. **c** Representative image of cleaved caspase of tumor tissues. **d** [^18^F]ML-10 uptake expressed as SUVmean in tumor control, tumor treated, and muscle at day 0, day 3, and day 6. Compared to control, ***p < 0.001 (Mann-Whitney test)

After two doses at a 72-h interval of 20 mg/kg paclitaxel, the volume of tumor was measured at days 0, 6, 10, 14, and 18, to evaluate the evolution of the tumor volume (Fig. 8a). Tumors got significantly reduced after paclitaxel treatment, from day 6 to the end of the study. [^18^F]ML-10 PET images were acquired at days 0, 3, and 6. At the baseline, we showed the radiotracer accumulation in the untreated tumor cells was higher compared to the muscle with a significant difference (*p* < 0.001; Additional file 1: Table S3). At the three time points of PET imaging, muscle uptake remained stable. At day 3, PET imaging data in the mice treated with paclitaxel demonstrated that tumor uptake was stable compared to the control mice (Fig. 8b–d). At day 6, [^18^F]ML-10 uptake decreased in the treated tumors (0.07 ± 0.02) compared to the control group (0.16 ± 0.02; *p* = 0.003), while increased binding was expected since the ex vivo analyses confirmed increased level of cleaved caspase 3 in the treated tumors, at d6 (Fig. 8c). However, [^18^F]ML-10 tumor uptake in both groups was always higher than the vascular background. Nevertheless, we showed that the radiotracer uptake in untreated tumors increased over time with a significant difference between day 0 (0.10 ± 0.03) and day 6 (0.16 ± 0.02; *p* = 0.005). The correlation analysis revealed a strong link between SUVmean and SUVmax ( Additional file 1: Figure S3a).

**Fig. 9.**
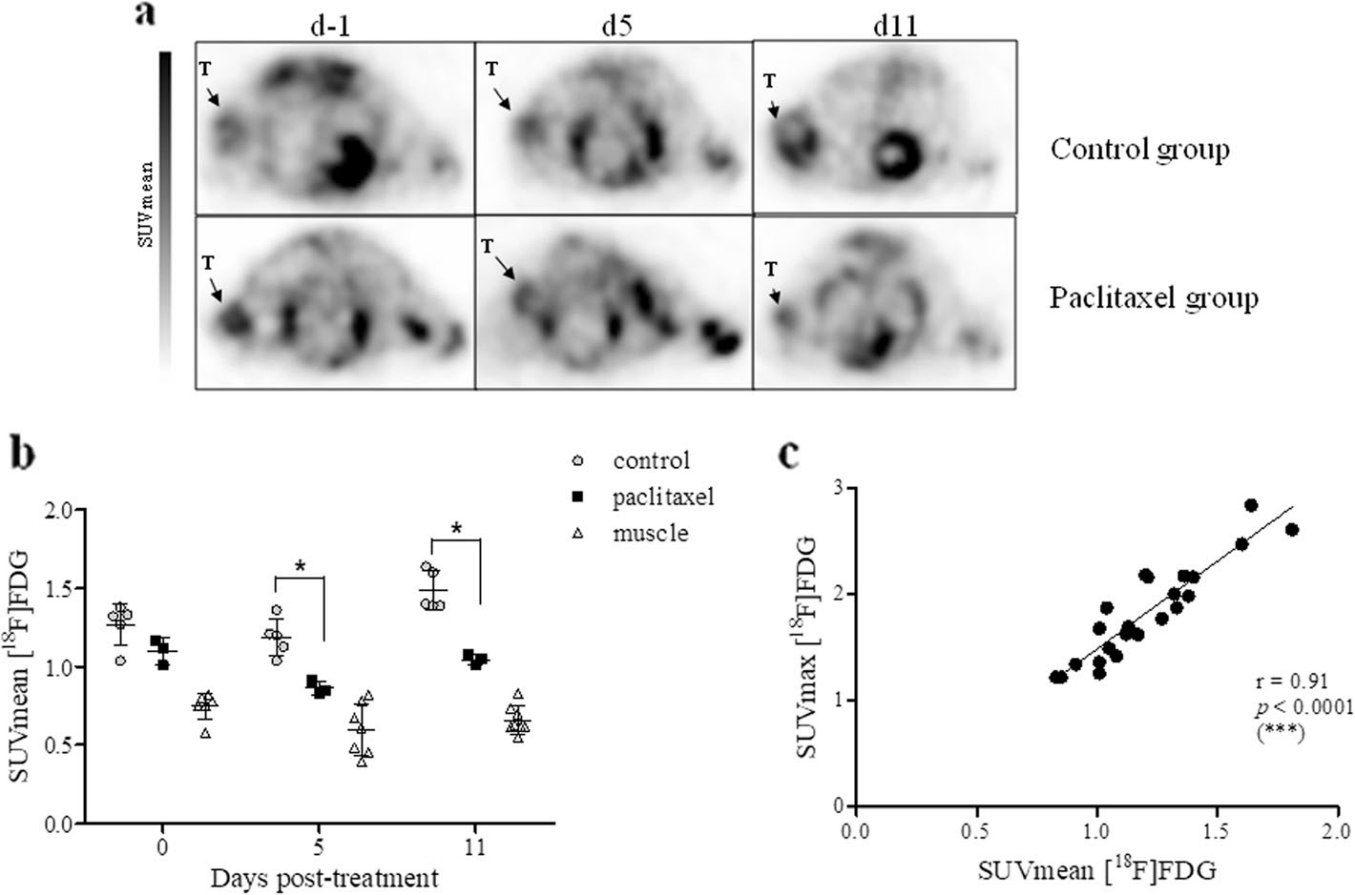
Evaluation of early response by [^18^F]FDG PET imaging on MDA-MB-468 subcutaneous model. **a** Images of [^18^F]FDG PET of representative MDA-MB-468 subcutaneous xenograft model performed and day 5 and day 11 post-treatment. T represented Tumor. **b** [^18^F]FDG uptake expressed as SUVmean in tumor control, tumor treated, and muscle before treatment and days 5 and 11 after treatment. Mann-Whitney test was performed (*p < 0.05). **c** Correlation between [^18^F]FDG uptake expressed by SUVmean and SUVmax. Pearson correlation was performed (***p < 0.001)

[^18^F]FDG PET images were acquired 1 day before treatment induction and then at day 5 and day 11 after treatment. Paclitaxel treatment induced a decrease of the [^18^F]FDG uptake at day 5 and day 11 (Fig. 9a). [^18^F]FDG SUVmean were respectively 0.86 ± 0.04; 1.04 ± 0.03 in the treated group compared to controls 1.19 ± 0.12; 1.48 ± 0.13 (*p* = 0.036 at day 5 and day 11; Fig. 9b; Additional file 1: Table S4). As expected, [^18^F]FDG tumoral SUVmean increased overtime in the control group (1.3 ± 0.1 at 5 day and 1.5 ± 0.1 at day 11 ; *p* = 0.008). SUVmean and SUVmax were strongly correlated (*p* < 0.001; Fig. 9c).

### Orthotopic model does not improve the predictive value of the [^18^F]ML-10 for early assessment of therapeutic response

**Fig. 10:**
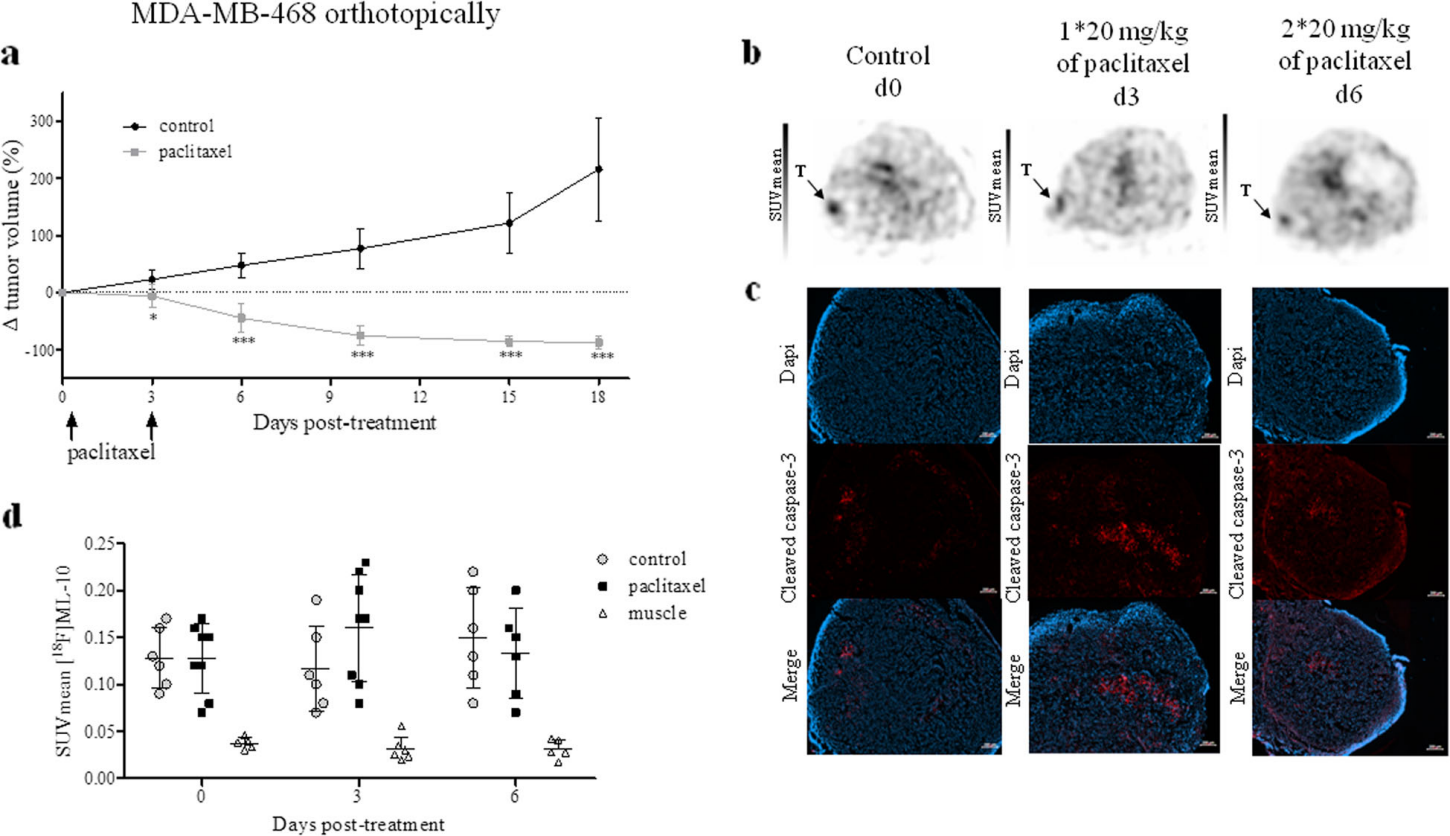
Evaluation of early response by [^18^F]ML-10 PET imaging on an orthotopic model. **a** Evaluation of tumor volume after two i.v. injections of paclitaxel (20 mg/kg) at a 72-h interval. Data are represented as mean ± SD. Compared to control, ***p < 0.001 (Mann-Whitney test). **b** Images of [^18^F]ML-10 PET of representative MDA-MB-468 orthotopic xenograft model performed and 72-h post-treatment. T represented tumor. **c** Representative image of cleaved caspase of tumor tissues. **d** [^18^F]ML-10 uptake expressed as SUVmean in tumor control, tumor treated and muscle at day 0, day 3, and day 6. Mann-Whitney test was performed (*p < 0.05)

The MDA-MB-468 orthotopic xenografts and the subcutaneous model responded to paclitaxel by a significant decrease of tumor volume from day 3 to the end of the study. Nevertheless, in the control groups, a difference in tumor growth was observed (Fig. 10a). The doubling time of the subcutaneous model was 16.4 days compared to 9.55 days for control tumors implanted in the mammary fat pad. In the non-treated models, [^18^F]ML-10 PET imaging demonstrated similar tumoral radiotracer accumulation irrespectively of the tumor location (Fig. 10b–d). In spite of apoptosis induction by paclitaxel, as demonstrated by increased cleaved caspase 3 expression in the treated tumors (Fig. 10c), no change in the radiotracer uptake was detected over the treatment time ( Additional file 1: Table S3). The SUVmean and SUVmax values highly positively correlated (*p* < 0.0001; Additional file 1: Figure S3b).

PET images generated from a dynamic PET experiment 45 min post injection confirmed a similar elimination of the radiotracer in non-target organs and tumor throughout the course of treatment ( Additional file 1: Figure S4).

## Discussion

Therapeutic strategies for TNBC need new methods of assessment of the tumor response to therapy, which could be combined with the currently used [^18^F]FDG PET imaging. The ultimate aim of such approaches is to achieve early evaluation of TNBC response to NAT (after only one or two therapy cycles), and a rapid change of the therapeutic protocols in case of tumor resistance. To our knowledge, the present report is the first to show the results of the evaluation of ^18^[F]ML-10, a proposed apoptosis radiotracer, as a tool for assessment of TNBC response to cytotoxic treatment in vitro and in vivo.

Following validation that paclitaxel induced apoptosis of TNBC cells in vitro, we showed that [^18^F]ML-10 uptake was increased, 72 h after treatment of MDA-MB-468 cells, and was associated with an increase of the apoptotic fraction. Furthermore, the high uptake was demonstrated in the adherent cells only (viable and early apoptotic) suggesting that the radiotracer is specific of early apoptosis, as previously proposed [18]. We did not confirm the absence of fixation of [^18^F]ML-10 to the necrotic cells. However, this was largely demonstrated by several teams [18, 19, 22]. Our results show, for the first time, that [^18^F]ML-10 indeed targets TNBC cells in early apoptosis after paclitaxel treatment in vitro.

In vivo, [^18^F]ML-10 distribution results were comparable to those obtained in humans with a very rapid clearance from non-target organs and a urinary elimination [19, 24]. Compared to other radiotracers targeting apoptosis (radiolabeled annexin V and Caspase3 radiolabeled), [^18^F]ML-10 PET presented a rapid distribution due to its low molecular weight, which allowed early tumor imaging after intravenous administration. In addition, its urinary elimination favored a better visualization of peritoneal tumors [17, 25]. Selective [^18^F]ML-10 uptake into specific tumor cells was observed and was more important in the MDA-MB-468 xenograft models compared to the MDA-MB-231 model. This accumulation was correlated with the basal apoptotic fraction determined by flow cytometry. It is interesting to note that a relation between tumor volume and radiotracer uptake was demonstrated in subcutaneous xenograft models but not in the orthotopic model. As already reported by several studies, the microenvironment of orthotopic implantation may be more favorable to tumor development [26].

In a second time, we performed the evaluation of early response to paclitaxel by [^18^F]ML-10 and [^18^F]FDG PET imaging on MDA-MB-468 xenograft models. The induction of apoptosis did not significantly correlate with the increase in [^18^F]-ML-10 uptake in the tumors. However, the [^18^F]FDG uptake reflected the decrease of tumor metabolism during the treatment. Similar findings were reported by other teams evaluating the early response with [^18^F]ML-10 in lymphoma model treated by cyclophosphamide [27].

Several hypotheses can explain the absence of correlation between [^18^F]ML-10 PET imaging and the early TNBC apoptosis, after exposure to chemotherapy. First, the delay between [^18^F]ML-10 imaging and the chemotherapy completion could have been non-optimal for visualization of apoptosis. However, we performed the PET imaging 72 h after treatment in accordance with the literature. In addition, the ex vivo analyses confirmed the increase of the apoptotic fraction at this time point. Secondly, the question of the impact of paclitaxel treatment on the in vivo distribution of the radiotracer was raised. However, dynamic PET images during treatment, on treated mice, did not show any difference in distribution of radiotracer in non-target organs. Thirdly, the sensitivity of the radiotracer must also be questioned. In our models, we were unable to detect, by PET imaging, the doubling of the apoptotic fraction after treatment. Nevertheless, before treatment, i.e., at the baseline, a positive correlation between the percentage of apoptotic cells and tumoral uptake of [^18^F]ML-10 before treatment was clearly evicence (*p* = 0.0008). Another important concern could be raised with regard to the method used for quantitative analysis of PET images, which we did not carry out. In clinical situations, a methodology based on voxel analysis has been developed [20]. It performs voxel-by-voxel subtraction of signal of pre-treatment from post-treatment and classifies voxels into three categories: those with positive accumulation, unchanged uptake or negative value to classify patients as responders or non-responders. This methodology has never been used in preclinical studies since it would require a highly reproducible registration of PET images and CT images. Finally, we assume that the most probable hypothesis that could explain the failure of [^18^F]ML-10 could be related to the pH variations of the tumor microenvironment during the chemotherapy. As shown in the literature, [^18^F]ML-10 is also a pH-sensitive radiotracer. Indeed, the malonic acid moieties of this radiotracer have pKa values of 3.07 and 5.78. At physiological pH, the di-anionic form of malonate predominates [20, 26, 28–30]. According to pKa values cited, a decrease of extracellular pH will favor the apparition of the mono-protonated carboxylic acid form of [^18^F]ML-10 and facilitate its interaction with the membrane of the apoptotic cells [30]. Hypoxia and apoptosis in solid tumors can lead to acidification of the intracellular milieu. In addition, some authors have demonstrated the ability of cancer cells to reverse the pH gradient across the membrane cell which could decrease the extracellular pH (acidification) and facilitate the [^18^F]ML-10 uptake [29, 31–33]. This hypothesis is supported by the correlation observed in our model between the fixation of [^18^F]ML-10 and [^18^F]FMISO. The in vitro results of radiotracer uptake reported herein and conducted with extracellular pH variations are consistent with such mechanism. Our hypothesis, confirmed by the literature, is that paclitaxel treatment of MDA-MB-468 xenograft models results in an increase of tumoral pH (basification) that will lead to a decrease of [^18^F]ML-10 cellular uptake. Nevertheless, this phenomenon was not observed in the orthotopic model. Zhang’s team was able to show that an orthotopic implantation revealed a more favorable and homogeneous growth of tumors as well as an increased microvessel density [26]. A better tumor vascularization then allows a better elimination of necrotic cells and therefore a less important variation in pH. However, even in this model, no significant increase in [^18^F]ML-10 tumor uptake was observed during apoptosis induced by treatment.

Our results demonstrated that [^18^F]ML-10 was not accurate to assess therapeutic response in chemotherapy setting probably due to the modification of extracellular pH conditions. However, the capacity of [^18^F]ML10 to predict the therapeutic response was confirmed in radiotherapy setting. The explanation is that radiotherapy treatment is known to induce, in addition to the “cell death” effect, inflammation at the tumor level that consequently modifies extracellular pH. Indeed, this inflammation leads to two phenomena: an activation of the immune system which will induce an increase in blood flow and an acidification of the microenvironment due to the release of immune cells and cytokines [34]. As a consequence, precedent clinical trials used [^18^F]ML-10 for PET imaging of apoptosis following radiotherapy treatments [21, 35].

## Conclusions

Our results in vitro confirmed that [^18^F]ML-10 specifically targets the cells in early apoptosis, after exposure of TNBC cell lines to paclitaxel for 72 h. In addition, favorable pharmacokinetic properties and high metabolic stability of this radiotracer were confirmed in basal apoptotic in vivo models. Nevertheless, we evidence that [^18^F]ML-10 did not allow imaging of in vivo apoptosis after paclitaxel treatment in a subcutaneous or orthotopic xenograft model of TNBC. Nevertheless, based on the several important issues highlighted in our study and taking into accounts the advantageous stability of [^18^F]ML-10 in basal conditions, this radiotracer may warrant further investigations in other preclinical cancer models than TNBC.

## Supplementary information

**Additional file 1.** Details about assessment of apoptosis and radiosyntheses of [^18^F]ML-10, [^18^F]FDG, and [^18^F]FMISO.

## Data Availability

Not applicable

## Abbreviations

[^18^F]FDG: 2-Deoxy-2-[^18^F]fluoro-d-glucose
[^18^F]FMISO: [^18^F]Fluoromisonidazole, 1-(2′-nitro-1′-imidazolyl)-3-[^18^F]fluoro-2-propranol
[^18^F]ML-10: 2-(5-[^18^F]fluoropentyl)-2-methyl-malonic acid
%IA/g: Percentage of injected activity per gram
CT: Computerized tomography
FEC: 5′-Fluorouracil + epirubicin + cyclophosphamide
FN: False negativity
FP: False positivity
IC_50_: Drug concentration that inhibited cell growth by 50%
i.v.: Intravenous injection
NAT: Neoadjuvant chemotherapy
pCR: Pathological complete response
PET: Positron emission tomography
PS: Phosphatidylserine
SD: Standard deviation
SPECT: Single photon emission computed tomography
SUV: Standard uptake value
TNBC: Triple negative breast cancer
TV: Tumor volume
HER2: Human epidermal growth factor receptor 2
