# Supplementary material for: [18F]ML-10 PET imaging fails to assess early response to neoadjuvant chemotherapy in a preclinical model of triple negative breast cancer
Source: EJNMMI Res. 2020 Jan 6;10:2. doi: 10.1186/s13550-019-0587-5 (PMC6944726; doi:10.1186/s13550-019-0587-5)
Supplement: Supplementary file 1 — Additional file 1. Details about assessment of apoptosis and radiosyntheses of [18F]ML-10, [18F]FDG, and [18F]FMISO. [file 13550_2019_587_MOESM1_ESM.docx]

**Online resource: Supplementary Material to**

**[^18^F]ML-10 PET imaging fails to assess early response to neoadjuvant chemotherapy in a preclinical model of triple negative breast cancer**

Elodie Jouberton^1,3,4^, Sébastien Schmitt^3,^ Emmanuel Chautard^2, 3^_,_ Aurélie Maisonial-Besset^3^, Marie Roy^3^, Nina Radosevic-Robin^2,3^, Jean-Michel Chezal^3^, Elisabeth Miot-Noirault^3^, Yann Bouvet^4^, Florent Cachin^1,3^

^1^ Service de Médecine Nucléaire, Centre Jean Perrin, Clermont-Ferrand, France

^2^ Département de Pathologie, Centre Jean Perrin, Clermont-Ferrand, France

^3^Université Clermont Auvergne, INSERM, Imagerie Moléculaire et Stratégies Théranostiques, UMR1240 Clermont-Ferrand, France

^4^Zionexa, Aubière, France

Correspondence to:

Florent Cachin

Centre de Lutte Contre le Cancer, Centre Jean Perrin, 58 rue Montalembert, 63011 Clermont-Ferrand

e-mail: florent.cachin@clermont.unicancer.fr

+334.73.27.80.81

***In vitro* characterization of chemotherapy-induced apoptosis**

**Cytotoxicity assays**

5 000 cells per well were seeded in 96-well plates for both cell lines and allowed to adhere overnight. Increasing paclitaxel and epirubicin concentrations diluted in sodium chloride (0.9% NaCl) were then added. After 72 hours of incubation, the cell viability was quantified by AlamarBlue assay. The cytotoxic activity was expressed as the drug concentration that inhibited cell growth by 50% (IC_50_). Experiments were performed in triplicates.

After determination of IC_50_ for each treatment in two human TNBC cell lines (MDA-MB-468 and MDA-MB-231), the apoptosis was induced in the same cell lines by incubating cells with paclitaxel or epirubicin (10 x IC_50_) in complete medium for 24 hours, 48 hours or 72 hours. The proportion of apoptotic cells was measured by flow cytometry (Annexin V / Propidium Iodide), western blotting (PARP activation) and Annexin V staining in real time.

**Flow cytometry**

After treatment, cells were washed with PBS 1X, harvested after trypsin-EDTA (Life Technologies) treatment and labeled using an Annexin V – FITC apoptosis detection kit (Miltenyi Biotec). Cells suspensions were analyzed by flow cytometry (LSR II flow cytometer, BD Biosciences). The analysis of the results was carried out using the BD FACS Diva^TM^ (BD Biosciences) software to determine the percentage of positive cells for Annexin V - FITC alone (apoptosis) or Annexin V - FITC and propidium iodide (necrosis). Experiments were performed in triplicates.

**IncuCyte apoptosis assays**

For apoptosis assays, Annexin V red reagent and Caspase 3/7 green reagent (IncuCyte, Essen Bioscience) were used following the manufacturer’s instructions. Fluorescence was measured one time per hour for 72 hours and were imaged with an IncuCyte imaging system (Essen Bioscience) at 37 °C, 5% CO_2_. Data were analyzed using the IncuCyte analysis software to detect and quantify the percentage of red cells (apoptotic) and were normalized to the well. The proliferation rate was assessed as changes in the area occupied by control or paclitaxel- or epirubicin-treated TNBC cells (% of confluence) over time. Experiments were performed in triplicates.

**Western blotting analyses**

For protein extraction, cells were washed with PBS, harvested and lysed using RIPA buffer supplemented with protease inhibitors (Protease inhibitor cocktail tablets, Roche^®^). On a 4-15% SDS gel (TGX gels, BioRad), 30 μg of protein extracts were separated. After blotting on a nitrocellulose membrane (45 μm, BioRad) nonspecific binding was blocked (5% non-fat milk) and membranes were incubated with specific primary antibodies PARP (Cell Signaling #9542), Caspase 3 (Cell Signaling #9668) or Actin (Sigma, A5441) at 4 °C, overnight. Antibody-antigen interactions were evidenced by chemiluminescence (Amersham) using HRP-conjugated secondary antibodies (Southern Biotech). The signal was detected using the Image Lab software (BioRad). Experiments were performed in triplicates.

**Evaluation of apoptosis in tumor**

Tumoral apoptosis was evaluated according to three different methods: western blotting (PARP, Caspase3), activated caspase 3 immunofluorescence and flow cytometry.

**Determination of the apoptotic markers by western blotting analyses**

Proteins were extracted from tissues by GentleMax tissue disruption in urea buffer supplemented with protease inhibitors (Protease inhibitor cocktail tablets, Roche^®^). Thirty microlitres of protein extracts were separated on a 4-15% SDS gel (TGX gels, BioRad). After blotting on a nitrocellulose membrane (45 μm, BioRad) nonspecific binding was blocked (5% non-fat milk) and membrane incubated with specific primary antibodies PARP (Cell Signaling #9542), Caspase 3 (Cell Signaling #9668) or Actin (Sigma, A5441) at 4 °C, overnight. Antibody-antigen interaction was evidenced by chemiluminescence (Amersham) using HRP-conjugated secondary antibodies (Southern Biotech). Signal was detected and quantified using the Image Lab software (BioRad). The ratio between the cleaved PARP protein and the total protein was calculated.

**Quantification of apoptotic cell by flow cytometry**

Fresh tumors were dissociated into single cell suspensions using Tumour Dissociation Kit (MACS, Miltenyi Biotec, Bergisch Gladbach, Germany) by mechanical dissociation and enzymatic degradation of the extracellular matrix. The analysis of tumor apoptosis was performed using a commercial kit (CaspGlowTM Fluorescein Active Caspase-3 Staining Kit, BioVision, Inc. USA) according to the manufacturer's protocol. Briefly, the single cells were incubated with DEVD-FMK conjugated with FITC, as a marker for direct determination of cleaved caspase 3 and incubated for 30 minutes at 37 °C in 5% CO_2_ atmosphere. After three cycles of washing with PBS 1X and centrifugation, cells were analyzed by flow cytometry (LSR II flow cytometer, BD Biosciences). The analysis of the results was carried out using the BD FACS Diva^TM^ (BD Biosciences) software to determine the percentage of positive cells for cleaved caspase 3.

**Assessment of apoptosis by immunohistochemistry to cleaved caspase 3**

Tumor tissues were embedded in tissue-Tek (OCT compound, Sakura-Finetek®), frozen at -80 °C and then 8 µm cryostat sections were cut and adhered to poly-L-Lysine-coated glass microscope slides. After fixation in 4% formalin (Sigma Aldrich, France), endogenous peroxidase quench and saturation, slides were incubated with the primary antibody: anti-Cleaved caspase 3 (1:200, Cell signaling #9664) rabbit polyclonal antibody at 4 °C overnight. Secondary antibody Alexa Fluor Rabbit 488 (Life Technologies, 1/200, 1 hour) was used to detect the primary antibody. Slides were washed with PBS 1X and mounted with Fluoroshield with Dapi (Sigma Aldrich, France) and glass coverslips.

**Synthesis of ML-10 and its radiolabeling precursor**

The ML-10 and its radiolabeling precursor were obtained as described in the literature[44] from di-*tert*-butyl malonate in six and four steps, respectively according to the synthesis scheme described below.

Reagents and conditions: (i) a) NaH, tert-butyl malonate, THF, 0 °C, 45 min then rt, 2 h; (b) benzyl 5-bromopent-1-yl oxide, 0 °C, then rt, 2 h followed by heating 50 °C, overnight; (ii) a) NaH, THF, 0 °C then rt, 1 h; (b) CH_3_I, 0 °C then rt, 15 h; (iii) H_2_, Pd/C 10%, CH_3_OH, rt, 19 h; (iv) MsCl, pyridine, rt, 4 h; (v) 1M TBAF, THF, CH_3_CN, reflux, 6 h 30; (vi) TFA, rt, 1 h.

*Material and methods*

All commercially available reagents and solvents were obtained from commercial sources (Aldrich, Acros) and used without purification. Benzyl 5-bromopent-1-yl oxide was purchased from ABCR GmbH. Unless otherwise noted, moisture sensitive reactions were conducted under dry argon atmosphere. Analytical thin layer chromatography (TLC) was performed on precoated silica gel 60 F_254_ plates (Macherey-Nagel) and visualized with UV light (254 nm) and/or developed with phosphomolybdic acid (8 wt%) in ethanol. Flash column chromatography was performed on silica gel 60A normal phase, 35–70 µm (Merck or SDS). Uncorrected melting points (mp) were recorded on an electrothermal capillary Digital Melting Point Apparatus IA9300 (Bibby Scientific). NMR spectra (200.13 MHz for ^1^H and 50.32 MHz for ^13^C J-Mod) were recorded on a Bruker Avance 200 instrument with chemical shift values (δ) expressed in parts per million (ppm) relative to residual solvent as standard and coupling constants (*J*) are given in Hz; ^19^F NMR spectra (470.6 MHz) were recorded on a Bruker Avance 500 apparatus using trifluorotoluene as internal reference (–63.72 ppm). Infrared spectra (IR) were recorded in the range 4000–440 cm^−1^ on a Nicolet IS10 (Fisher Scientific) with attenuated total reflectance (ATR) accessory. Compounds were analyzed by High-Resolution Mass Spectrometry (HRMS) in positive or negative mode (ThermoScientific^®^ Orbitrap Q-Exactive).

*di-tert-butyl 2-[(5-benzyloxy)pent-1-yl]malonate (****1****)*

To a suspension of sodium hydride (60 wt% dispersion in mineral oil, 194 mg, 4.85 mmol) in anhydrous tetrahydrofuran (20 mL) at 0 °C was added di-tert-butyl malonate (1 mL, 4.47 mmol). The solution was stirred at 0 °C for 45 minutes then at room temperature for 2 hours. After cooling down to 0 °C, a solution of benzyl 5-bromopent-1-yl oxide (741 μL, 3.57 mmol) in anhydrous tetrahydrofuran (15 mL) was added dropwise over 30 minutes. The reaction mixture was warmed to room temperature, stirred for 2 hours (the solution turned milky) then at 50 °C overnight. After cooling down to room temperature, brine (45 mL) was added and the mixture was extracted with diethyl ether (3 x 75 mL). The combined organic layers were dried over magnesium sulfate, filtered and evaporated under reduced pressure. The crude was purified by column chromatography (SiO_2_, cyclohexane/diethyl ether 95/5 then 5/5, v/v) to give compound **1** as a colourless oil (1.10 g, 2.80 mmol). Yield: 78%. R*_f_* = 0.10 (SiO_2_, cyclohexane/diethyl ether 95/5, v/v); IR (ATR accessory) ν 2977, 2934, 1367, 1251, 1137 cm^-1^; ^1^H NMR (200.13 MHz, CDCl_3_)  1.25-1.50 (m, 22H), 1.60 (m, 2H), 1.80 (m, 2H), 3.10 (t, 1H, *J* = 7.5 Hz), 3.45 (t, 2H, *J*= 6.3 Hz), 4.48 (s, 2H), 7.00-7.35 (m, 5H); ^13^C NMR (50.32 MHz, CDCl_3_) δ 26.0, 27.1, 28.0 (6C), 25.6, 29.6, 54.0, 70.3, 72.9, 81.2 (2C), 127.5, 127.6 (2C), 128.4 (2C), 138.7, 169.0 (2C); HRMS (ESI) calculated for C_23_H_35_O_5_ [M-H]^-^ requires 391.2479, found 391.2479.

*di-tert-butyl 2-[(5-benzyloxy)pent-1-yl]-2-methylmalonate (****2****)*

To a suspension of sodium hydride (60 wt% dispersion in mineral oil, 878 mg, 22.0 mmol) in anhydrous tetrahydrofuran (40 mL) at 0 °C was added a solution of ester **1** (4.31 g, 11.0 mmol) in anhydrous tetrahydrofuran (40 mL). The solution was warmed to room temperature and stirred for 1 hour. After cooling down to 0 °C, methyl iodide (6.84 mL, 110 mmol) was slowly added. The flask was sealed with a glass stopper and the reaction was stirred at room temperature for 15 hours. After cooling down to 0 °C, brine (60 mL) was added and the solution was extracted with diethyl ether (3 x 80 mL). The combined organic layers were dried over magnesium sulfate, filtered and evaporated under reduced pressure. The crude was purified by column chromatography (SiO_2_, cyclohexane/diethyl ether 95/5, v/v) to give compound **2** as a colourless oil (3.91 g, 9.62 mmol). Yield: 87%. R*_f_* = 0.15 (SiO_2_, cyclohexane/diethyl ether 95/5, v/v); IR (ATR accessory) ν 2977, 2933, 2858, 1725, 1367, 1253, 1157, 1119 cm^-1^; ^1^H NMR (200.13 MHz, CDCl_3_) δ 1.15-1.35 (m, 5H), 1.35-1.50 (m, 20H), 1.61 (m, 2H), 1.77 (m, 2H), 3.44 (t, 2H, *J* = 6.3 Hz), 4.48 (s, 2H), 7.20-7.40 (m, 5H); ^13^C NMR (50.32 MHz, CDCl_3_) δ 19.7, 24.1, 26.7, 27.9 (6C), 29.6, 35.4, 54.6, 70.3, 72.9, 80.8 (2C), 127.5, 127.6 (2C), 128.4 (2C), 138.7, 171.9 (2C); HRMS (ESI) calculated for C_24_H_37_O_5_ [M-H]^-^ requires 405.2636, found 405.2636.

*di-tert-butyl 2-(5-hydroxypent-1-yl)-2-methylmalonate (****3****)*

A mixture of benzyl derivative **2** (2.00 g, 4.92 mmol) and Pd/C 10% (104 mg) in methanol (45 mL) was stirred under a hydrogen atmosphere at room temperature for 19 hours. The reaction mixture was filtered over celite 545 bed, which was then washed with methanol (2 x 10 mL). The filtrate was evaporated under reduced pressure and the residue was purified by column chromatography (SiO_2_, ethyl acetate/cyclohexane 3/7 then 5/5, v/v) to give compound **3** as a colourless oil (1.40 g, 4.42 mmol). Yield: 90%. R*_f_* = 0.72 (SiO_2_, ethyl acetate/cyclohexane 5/5, v/v); IR (ATR accessory) ν 3600-3150, 2978, 2935, 2864, 1723, 1368, 1252, 1152, 1117 cm^-1^; ^1^H NMR (200.13 MHz, CDCl_3_) δ 1.10-1.60 (m, 27H), 1.72 (m, 2H), 3.59 (t, 2H, *J*= 6.3 Hz); ^13^C NMR (50.32 MHz, CDCl_3_) δ 19.8, 24.0, 26.2, 28.0 (6C), 32.6, 35.4, 54.7, 62.9, 81.0 (2C), 172.0 (2C).

*di-tert-butyl 2-methyl-2-[(5-methylsulfonyloxy)pent-1-yl]malonate (****4****)*

 To a solution of alcohol **3** (0.70 g, 2.21 mmol) in anhydrous pyridine (4 mL) was added under argon methanesulfonyl chloride (514 μL, 6.64 mmol). The reaction mixture was stirred at room temperature for 4 hours. The solution was quenched with saline (20 mL) at 0 °C then warmed to room temperature and extracted with diethyl ether (3 x 30 mL). The combined organic layers were dried over magnesium sulfate, filtered and evaporated under reduced pressure. The crude was purified by column chromatography (SiO_2_, dichloromethane) to give radiolabelling precursor **4** as a colorless oil (439 mg, 1.11 mmol). Yield: 50%. R*_f_* = 0.38 (SiO_2_, dichloromethane); IR (ATR accessory) ν 2976, 2936, 1721, 1355, 1255, 1171, 1119 cm^-1^; ^1^H NMR (200.13 MHz, CDCl_3_) δ 1.20-1.35 (m, 5H), 1.35-1.55 (m, 22H), 1.77 (m, 2H), 2.99 (s, 3H), 4.21 (t, 2H, *J* = 6.4 Hz); ^13^C NMR (50.32 MHz, CDCl_3_) δ 19.7, 23.6, 25.8, 27.8 (6C), 28.8, 35.1, 37.3, 54.4, 69.9, 80.9 (2C), 171.6 (2C).

di-*tert*-Butyl 2-(5-fluoropent-1-yl)-2-methylmalonate (**5**)

 To a solution of mesylate **4** (439 mg, 1.11 mmol) in anhydrous acetonitrile (20 mL) was added dropwise at room temperature a solution of 1 M tetrabutylammonium fluoride in tetrahydrofuran (1.34 mL, 1.34 mmol). The reaction mixture was refluxed for 4 h 30. TLC analysis indicated an incomplete reaction. Additional 1 M tetrabutylammonium fluoride in tetrahydrofuran (0.60 mL, 0.60 mmol) was then added and the reaction was refluxed for 2 h. After cooling down to room temperature, water (40 mL) was added and the mixture was extracted with diethyl ether (3 x 40 mL). The combined organic layers were dried over magnesium sulfate, filtered and evaporated under reduced pressure. The crude was purified by column chromatography (SiO_2_, cyclohexane/ethyl acetate 97/3, v/v) to give compound **5** as a colorless oil (214 mg, 0.67 mmol). Yield: 60%. R*_f_* = 0.79 (SiO_2_, cyclohexane/ethyl acetate 97/3, v/v); IR (ATR accessory) ν 2977, 2936, 1724, 1368, 1254, 1156, 1119 cm^-1^; ^1^H NMR (200.13 MHz, CDCl_3_)  1.15-1.35 (m, 5H), 1.35-1.50 (m, 20H), 1.61 (m, 2H), 1.76 (m, 2H), 4.40 (dt, 2H, *J* = 5.9 Hz, ^2^*J*_H-F_ = 47.3 Hz); ^13^C NMR (50.32 MHz, CDCl_3_) δ 19.8, 23.9, 25.7 (d, *^3^J*_C-F_ = 5.2 Hz), 28.0 (6C), 30.3 (d, ^2^*J*_C-F_ = 19.4 Hz), 35.3, 54.6, 81.0 (2C), 84.0 (d, ^1^*J*_C-F_ = 164.3 Hz), 171.9 (2C); ^19^F NMR (470.6 MHz, CDCl_3_) δ 219.4.

*2-(5-Fluoropent-1-yl)-2-methylmalonic acid (****ML-10****)*

 A solution of ester **5** (70 mg, 0.22 mmol) in trifluoroacetic acid (2 mL) was stirred at room temperature for 1 h. The reaction mixture was evaporated under reduced pressure to give ***ML-10*** as a white precipitate (42 mg, 0.20 mmol). Yield: 93%. mp 111 °C (Litt. 103-105 °C[44]); R*_f_* = 0.79 (SiO_2_, cyclohexane/ethyl acetate 97/3, v/v); IR (ATR accessory) ν 3200-2700, 2700-2400, 1698, 1280, 1261 cm^-1^; ^1^H NMR (200.13 MHz, CDCl_3_)  1.15-1.55 (m, 7H), 1.65 (m, 2H), 1.92 (m, 2H), 4.44 (dt, 2H, *J* = 5.8 Hz, ^2^*J*_H-F_ = 47.2 Hz), 10.0 (brs, 2H); ^13^C NMR (50.32 MHz, CDCl_3_) δ 20.0, 24.1, 25.5 (d, *^3^J*_C-F_ = 4.9 Hz), 30.2 (d, ^2^*J*_C-F_ = 19.6 Hz), 35.6, 53.9, 84.0 (d, ^1^*J*_C-F_ = 164.2 Hz), 178.1 (2C); ^19^F NMR (470.6 MHz, CDCl_3_) δ 219.4. ESI-MS m/z 205.06 [M-H]^+^. HRMS (ESI) calculated for C_9_H_14_O_4_F [M-H]^-^ requires 205.0871, found 205.0871.

**Production of fluoride-18**

No-carrier-added fluorine-18 (half-life: 109.8 min) was produced via the [^18^O(p, n)^18^F] nuclear reaction by irradiation of a 2.8 mL >97%-enriched [^18^O]H_2_O target (Bruce Technology) on a CPH14 cyclotron (14 MeV proton beam, Currium laboratories).

**Radiosynthesis of [^18^F]ML-10, [^18^F]FDG and [^18^F]FMISO**

[^18^F]FDG was purchased from commercially available source (Cyclopharma S.A., Janneyrias, France).

Radiochemical syntheses and semi-preparative HPLC purifications were performed using a mono-reactor SynChrom R&D EVOI syntheses module (Raytest).

***Radiolabeling of [^18^F]ML-10***

[^18^F]fluoride (15 - 25 GBq) in [^18^O]H_2_O (3.8 mL) was trapped on QMA light cartridge (46 mg, Waters, trap 1) to remove [^18^O]H_2_O and further eluted in the reaction vessel with a solution of potassium carbonate (2.1 mg, 15 µmol) and Kryptofix 2.2.2 (7 mg, 18.5 µmol) in a mixture acetonitrile/water (1 mL, 7/3, v/v, vial 1). The mixture was heated at 100 °C for 3 minutes under reduced pressure and helium flow and cooled to 25 °C. Anhydrous acetonitrile (1 mL, vial 2) was added and the mixture heated at 110 °C for 3 minutes under reduced pressure and helium flow and cooled to 25 °C to give anhydrous K[^18^F]/K2.2.2/carbonate complex. The mesylate labeling precursor 4 (4 mg, 10 µmol) in anhydrous acetonitrile (1 mL, vial 3) was added and the mixture heated at 90 °C for 15 min. The mixture was cooled to 40 °C and an aqueous 3 N HCl solution (0.5 mL, vial 4) was added. The mixture was heated at 115 °C for 15 minutes. After cooling down to 40 °C, the reaction mixture was quenched with an aqueous mixture of 1 M NaOH/0.25 M AcONa (1 mL, vial 5) and injected into a semi-preparative HPLC system (XBridge® Prep C18 5µm 10 x 250 mm, mobile phase: water/acetonitrile/acetic acid, 70/30/0.5, v/v/v, 3 mL/min, t_R_ = 6 min). The collected fraction was diluted in water (60 mL) and passed through an Oasis Plus HLB cartridge (Water, trap 3). The cartridge was washed with water (10 mL, vial 9) and eluted with ethanol (2.5 mL, vial 7). The eluted ethanolic solution was then concentrated under reduced pressure and diluted in sterile saline (0.9% NaCl, 1 mL) to give pure [^18^F]ML-10 usable for injection. The radiosynthesis was performed in 110-130 minutes including formulation, and [^18^F]ML-10 was obtained with radiochemical yields of 23.3 ± 10.8% (n = 28, decay corrected). Typically, 1-2.5 GBq of radiotracer were obtained starting from 15-25 GBq of [^18^F]fluoride.

***Quality control of [^18^F]ML-10***

Analytical HPLC measurements were performed on a system consisting of an Agilent HP series 1100 (Hewlett Packard, Les Ulis, France) combined with a Flow one A500 Radiomatic detector (Packard, Canberra, Australia). An aliquot (1-2 µL, 55-74 kBq) of the [^18^F]ML-10 solution diluted in water (20 µL) was injected into the analytical HPLC system mentioned above (Agilent Zorbax extend C18 column, 4.6 mm × 150 mm, 5 µm), using water/acetonitrile/TFA (70/30/0.1, v/v/v, 1 mL/min, t_R_ = 4.20 min) as eluent. [^18^F]ML-10 was obtained with radiochemical purity > 99%.

Aliquot (1-2 µL, 55-74 kBq) was spotted on a TLC plate (silica gel, 60 F254; Alugram XTra G/UV254; Macherey-Nagel). TLC plate was eluted with a mixture of acetonitrile/water (95/5, v/v) and analyzed with a Minigita DUAL radio-TLC scanner (Elysia-Raytest, PET probe).

***Radiolabeling of [^18^F]FMISO***

[^18^F]fluoride (15 - 25 GBq) in [^18^O]H_2_O (3.8 mL) was trapped on QMA light cartridge (46 mg, Waters, trap 1) to remove [^18^O]H_2_O and further eluted in the reaction vessel with a solution of potassium carbonate (3 mg, 21 µmol) and Kryptofix 2.2.2 (15 mg, 40 µmol) in a mixture acetonitrile/water (1 mL, 7/3, v/v, vial 1). The mixture was heated at 100 °C for 3 minutes under reduced pressure and helium flow and cooled to 25 °C. Anhydrous acetonitrile (1 mL, vial 2) was added and the mixture was heated at 110 °C for 3 minutes under reduced pressure and helium flow and cooled to 25 °C to give anhydrous K[^18^F]/K2.2.2/carbonate complex. NITTP (ABX, 5 mg, 11.8 µmol) in anhydrous acetonitrile (1 mL, vial 3) was added and the mixture was heated at 95 °C for 10 minutes and cooled to 25 °C. The mixture was heated at 110 °C for 2 minutes under reduced pressure to remove acetonitrile, cooled to 25°C and 0.5 N HCl (1 mL, vial 4) was added. The mixture wad heated at 100 °C for 4 minutes. After cooling down to 25 °C, the reaction mixture was quenched with 30% AcONa (1.5 mL, vial 5) and injected into a semi-preparative HPLC system (SymmetryPrep C18, 7µm 7.8 x 300 mm, mobile phase : water/ethanol, 95/5, v/v, 3 mL/min, t_R_ = 11-12 min). The collected fraction contained pure [^18^F]FMISO directly usable for injection. The radiosynthesis was performed in 60-70 minutes including formulation, and [^18^F]FMISO was obtained with radiochemical yields of 26.8 ± 4.9% (n = 8, decay corrected). Typically, 2 - 4.5 GBq of radiotracer were obtained starting from 15-25 GBq of [^18^F]fluoride.

***Quality control of [^18^F]FMISO***

Analytical HPLC measurements were performed on a system consisting of an Agilent HP series 1100 (Hewlett Packard, Les Ulis, France) combined with a Flow one A500 Radiomatic detector (Packard, Canberra, Australia). An aliquot (1-2 µL, 55-74 kBq) of the [^18^F]FMISO solution diluted in water (200 µL) was injected into the analytical HPLC system mentioned above (Agilent Zorbax extend C18 column, 4.6 mm × 150 mm, 5 µm), using water/ethanol (linear gradient from 2% to 10% of ethanol for 10 min, 1 mL/min, t_R_ = 6.8 min) as eluent. [^18^F]FMISO was obtained with radiochemical purity > 99%.

**TABLE**

**Table S1:** Comparison of [^18^F]ML-10 tumor uptake in subcutaneously MDA-MB-231 xenograft, subcutaneously and orthotopic MDA-MB-468 xenograft models.

|  | **[^18^F]ML-10** | | |
| --- | --- | --- | --- |
|  | SUVmean | SUVmax | T/M |
| **Subcutaneously MDA-MB-231** *(n = 28)* | 0.07 ± 0.03 | 0.15 ± 0.07 | 2.33 ± 1.71 |
| **Subcutaneously MDA-MB-468** *(n = 28)* | 0.12 ± 0.03 | 0.26 ± 0.05 | 6.69 ± 2.94 |
| **Orthotopically MDA-MB-468** *(n = 27)* | 0.12 ± 0.04 | 0.26 ± 0.13 | 3.11 ± 1.37 |
| **Muscle** *(n = 27)* | 0.03 ± 0.01 | 0.07 ± 0.03 | / |

Data are presented as the mean ± SD.

**Table S2:** Comparison of [^18^F]ML-10, [^18^F]FDG and [^18^F]FMISO tumor uptake in subcutaneously MDA-MB-231 xenograft, subcutaneously and orthotopic MDA-MB-468 xenograft models.

|  | **[^18^F]FDG** | | |  | **[^18^F]FMISO** | | |
| --- | --- | --- | --- | --- | --- | --- | --- |
|  | SUVmean | SUVmax | T/M |  | SUVmean | SUVmax | T/M |
| **Subcutaneously MDA-MB-468**  *(n = 12)* | 1.28 ± 0.31 | 1.98 ± 0.5 | 1.76 ± 0.7 |  | 0.60 ±0.18 | 1.59 ± 0.50 | 13.20 ± 10.16 |
| **Orthotopically MDA-MB-468**  *(n = 9)* | 1.33 ± 0.36 | 1.76 ± 1.54 | 2.71 ± 0.98 |  | 0.81 ± 0.26 | 1.50 ± 0.43 | 16.12 ± 6.48 |

Data are presented as the mean ± SD.

**Table S3.** Comparison of variation change in tumor volume and tumor uptake [^18^F]ML-10 at day 0, day 3 and day 6 on MDA-MB-468 xenograft models.

|  |  |  |  | **[^18^F]ML-10** | | |  |
| --- | --- | --- | --- | --- | --- | --- | --- |
|  | **Groups** | **Days after treatment** | **Δ TV** | **SUVmean** | **SUVmax** | **T/M** | |
| **Subcutaneously MDA-MB-468** | Control  *(n= 7)* | 0 | - | 0.10 ±0.03 | 0.26 ± 0.11 | 3.7 ± 1.2 | |
|  |  | 3 | 36.8 ± 23.5 | 0.14 ± 0.03 | 0.26 ± 0.03 | 2.8 ± 1.2 | |
|  |  | 6 | 92.6 ± 36.1 | 0.16 ± 0.02^##^ | 0.28 ± 0.03 | 5.2 ± 2.1 | |
|  |  |  |  |  |  |  | |
|  | Paclitaxel  20 mg/kg *(n=6)* | 0 | - | 0.12 ± 0.02 | 0.19 ± 0.01 | 4.69 ± 1.5 | |
|  |  | 3 | 8.91 ± 21.6 | 0.15 ± 0.02 | 0.33 ± 0.12 | 4.70 ± 2.9 | |
|  |  | 6^a^ | -29.8 ± 18.6 ^***^ | 0.07 ± 0.02 ^**^ | 0.15 ± 0.05 ^**^ | 1.59 ± 0.7 ^**^ | |
| **Orthotopically MDA-MB-468** | Control  *(n=6)* | 0 | - | 0.13 ± 0.03 | 0.23 ± 0.06 | 2.6 ±0.7 | |
|  |  | 3 | 22.9 ± 17.9 | 0.12 ± 0.05 | 0.26 ± 0.05 | 3.8 ± 0.8 | |
|  |  | 6 | 47.7 ± 31.1 | 0.15 ± 0.05 | 0.35 ± 0.07 | 4.1 ± 0.9 | |
|  |  |  |  |  |  |  | |
|  | Paclitaxel  20 mg/kg *(n=8)* | 0 | - | 0.13 ± 0.04 | 0.35 ± 0.17 | 2.8 ± 1.5 | |
|  |  | 3 | -6.25 ± 20.1 ^*^ | 0.16 ± 0.06 | 0.39 ± 0.14 | 4.3 ± 1.9 | |
|  |  | 6^a^ | -44.1 ± 25.4 ^***^ | 0.13 ± 0.05 | 0.32 ± 0.21 | 4.2 ± 1.8 | |

Data are presented as the mean ± SD. ^a^ Corresponding 72h after second dose of paclitaxel. ^*^ Significant difference vs control. ^#^ Significant difference vs day 0.

**Table S4.** Comparison of variation change in tumor volume and tumor uptake [^18^F]FDG at day 0, day 5 and day 11 on MDA-MB-468 subcutaneous xenograft model.

|  |  |  |  | **[^18^F]FDG** | | |  |
| --- | --- | --- | --- | --- | --- | --- | --- |
|  | **Groups** | **Days after treatment** | **Δ TV** | **SUVmean** | **SUVmax** | **T/M** | |
| **Subcutaneously MDA-MB-468** | Control  *(n= 7)* | 0 | - | 1.27 ± 0.13 | 2.05 ± 0.33 | 1.81 ± 0.58 | |
|  |  | 3 | 36.8 ± 23.5 | 1.19 ± 0.12 | 2.01 ± 0.22 | 1.47 ± 0.43 | |
|  |  | 6 | 92.6 ± 36.1 | 1.48 ± 0.13^##^ | 2.49 ± 0.34 | 2.53 ± 0.55 | |
|  |  |  |  |  |  |  | |
|  | Paclitaxel  20 mg/kg *(n=6)* | 0 | - | 0.94 ± 0.46 | 1.50 ± 0.21 | 1.25 ± 0.24 | |
|  |  | 3 | 8.91 ± 21.6 | 0.78 ± 0.39^*^ | 1.26 ± 0.07* | 1.73 ± 0.69 | |
|  |  | 6^a^ | -29.8 ± 18.6 ^***^ | 0.95 ± 0.49^*^ | 1.42 ±0.07 | 1.27 ± 0.55 | |

Data are presented as the mean ± SD. * Significant difference vs control. ^a^ Corresponding 72h after second dose of paclitaxel. ^#^ Significant difference vs day 0.

**FIGURES**


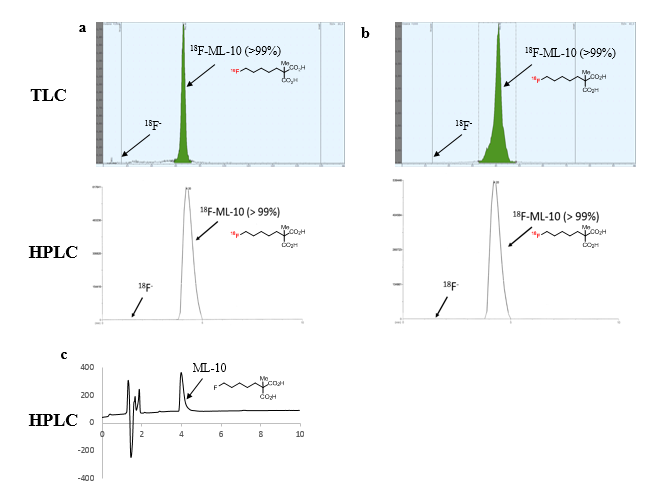


**Fig. S1. Representative radio-TLC and radio-HPLC chromatograms of [^18^F]ML-10** after formulation (**a**), and in urine samples (**b**) taken 60 min after i.v. injection of [^18^F]ML-10 into a mouse. The radio-HPLC retention time of [^18^F]ML-10 was 4.20 minutes. (**c**) Representative HPLC chromatogram of non-radioactive reference (λ = 210 nm, t_R_ = 3.98 minutes).

**
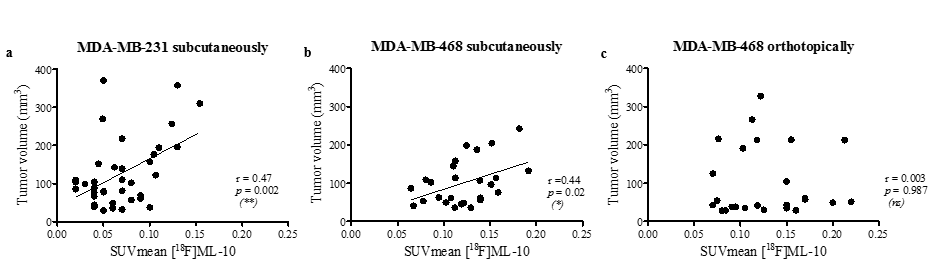
**

**Fig. S2.** Correlation analysis between tumor volume and SUVmean of [^18^F]ML-10 in (**a**) MDA-MB-231 subcutaneous xenograft model, (**b**) MDA-MB-468 subcutaneous xenograft model and (**c**) MDA-MB-468 orthotopic xenograft model. Pearson correlation was performed (*p < 0.05, **p < 0.01).


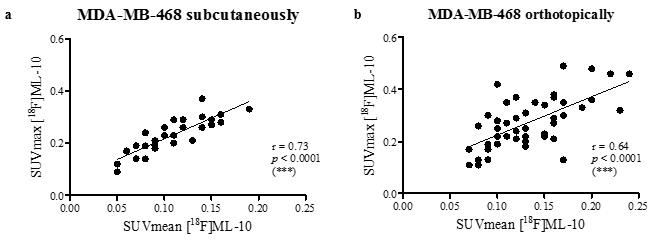


**Fig. S3.** Correlation between [^18^F]ML-10 uptake expressed by SUVmean and SUVmax. in (**a**) MDA-MB-468 subcutaneous xenograft model, (**b**) MDA-MB-468 orthotopic xenograft model. Pearson correlation was performed (***p < 0.001).

**
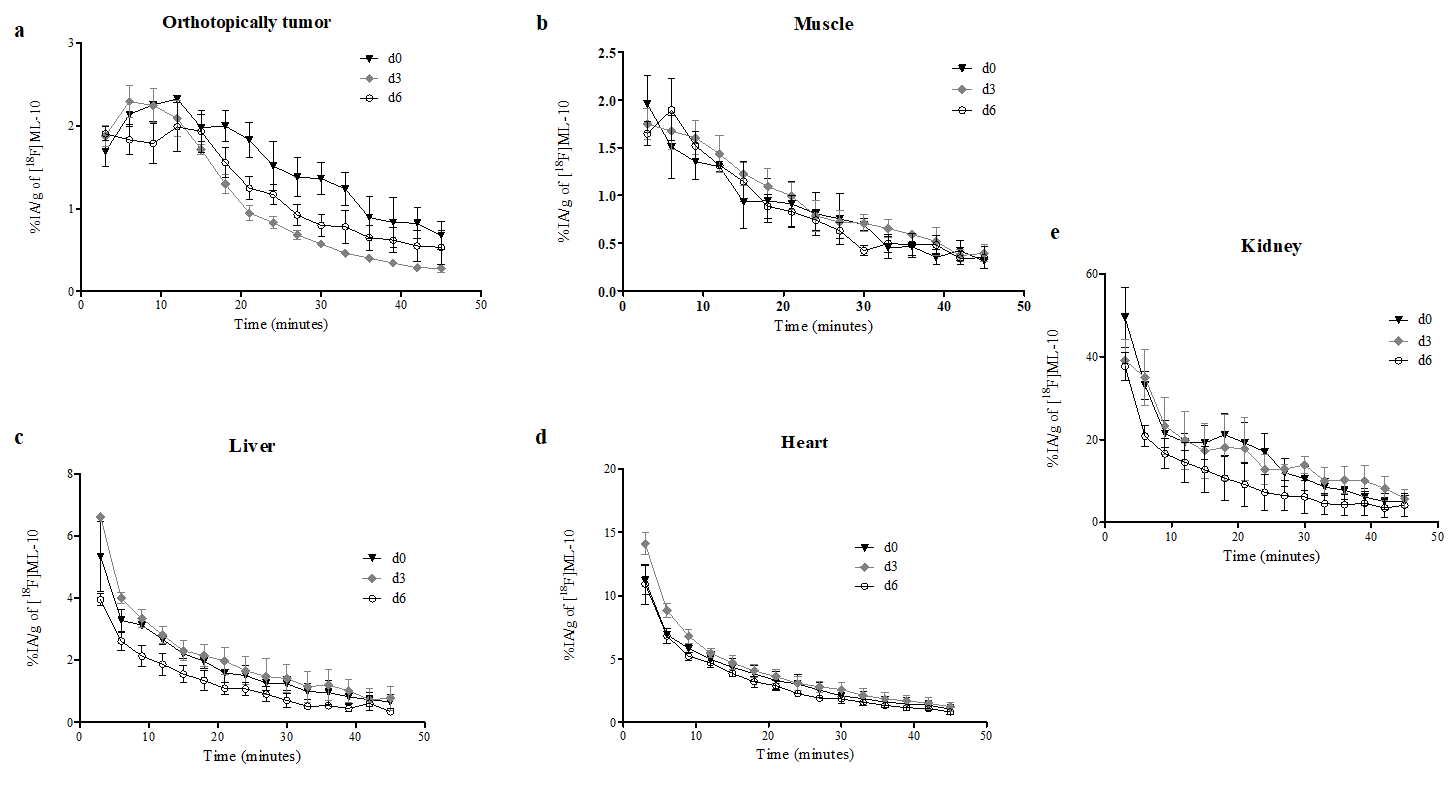
**

**Fig. S4.** Time activity curves after bolus injection of 29.5 ± 4.1 MBq [^18^F]ML-10 in triple negative breast cancer model during treatment. Time activity curves showing uptake of [^18^F]ML-10 in MDA-MB-468 orthotopic tumor (a), muscle (b), liver (c), heart (d) and kidney (e) over 45 min (n=3). Subsequent [^18^F]ML-10 PETs were performed before treatment (d0) and 72 hours after each treatment dose (d3 and d6). Radioactivity was expressed as a percentage of the injected activity per gram of tissue (%IA/g). Data are presented as mean ± SD.”


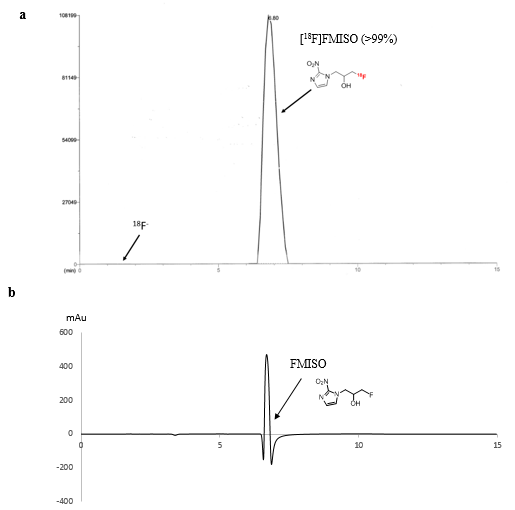


**Fig. S5.** Representative radio-HPLC chromatogram of [^18^F]FMISO obtained after formulation (**a**) (t_R_ = 6.80 minutes) compared to UV-HPLC chromatogram of its non-radioactive reference (**b**) (λ = 250 nm, t_R_ = 6.69 minutes).
